# Electron‐Beam Enables Spatially Resolved Defect‐Mediated Work Function Modulation in FAPbI_3_ Perovskite Thin Films

**DOI:** 10.1002/advs.77723

**Published:** 2026-09-16

**Authors:** Hyeonho Park, Seung‐Gu Choi, Jae‐Hwan Kim, Dong‐Jun Lee, Sung‐Kwang Jung, Gwanghee Lee, HongSeok Kim, Kyung‐Hwan Jin, Jin‐Wook Lee, Seong Heon Kim

**Affiliations:** ^1^ Department of Physics Jeonbuk National University Jeonju Republic of Korea; ^2^ Park Systems Corp. Gwacheon Republic of Korea; ^3^ Department of Nano Engineering and Department of Nano Science and Technology SKKU Advanced Institute of Nanotechnology (SAINT) Sungkyunkwan University Suwon Republic of Korea; ^4^ Research Institute of Energy and Resources Seoul National University Seoul Republic of Korea; ^5^ Department of Energy Systems Engineering College of Engineering Seoul National University Seoul Republic of Korea; ^6^ Davidson School of Chemical Engineering Purdue University West Lafayette Indiana USA; ^7^ Research Institute For Materials and Energy Science Jeonbuk National University Jeonju Republic of Korea; ^8^ School of Transdisciplinary Innovations Seoul National University Seoul Republic of Korea

**Keywords:** defects, electron beam, FAPbI_3_, irradiation, perovskites

## Abstract

Modulation of the electronic energy levels in semiconducting materials is an essential technology for fabrication of advanced optoelectronic devices. Despite active studies on metal halide perovskite materials, a versatile modulation methodology capable of spatially uniform and controllable adjustment of the Fermi level remains elusive. Herein, we demonstrate that electron beam irradiation enables systematic tuning of work function in widely used formamidinium lead triiodide (FAPbI_3_) perovskite thin films. The electron‐beam irradiation was found to induce iodine‐rich surface defect states, including interstitial iodine/polyiodide‐like species, thereby reducing the electron density near the Pb‐I coordination sites and leading to acceptor‐like surface electronic modulation. Variations in acceleration voltage and exposure time produced systematic work‐function modulation, reflecting the combined influence of incident electron energy and total electron dose on iodine‐related defect redistribution. Exploiting spatial controllability and local work function modulation, a lateral built‐in potential of 0.62 V in FAPbI_3_ thin films was achieved. The proof‐of‐concept device based on the surface‐potential‐defined lateral junction formed by electron‐beam irradiation exhibited clear rectifying current–voltage behavior.

## Graphical abstract

Electron‐beam irradiation enables defect‐mediated work function modulation in metal halide perovskites by regulating interstitial iodides. This approach allows continuous Fermi‐level tuning and spatially resolved formation of lateral surface‐potential‐defined junctions with built‐in potential.

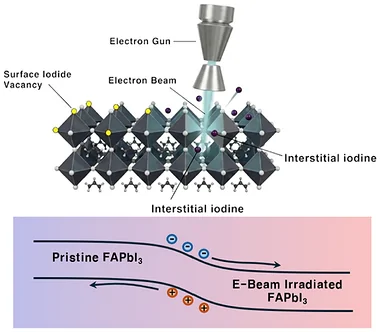

## 1 Introduction

Organometallic halide perovskites (OHPs) have emerged as one of the most promising classes of semiconductors for next‐generation optoelectronic technologies, owing to their exceptional optoelectronic properties, including strong light absorption, long carrier diffusion lengths, and intrinsic defect tolerance, together with a highly tunable bandgap [1, 2, 3]. Since their initial success in photovoltaic applications, halide perovskites have rapidly expanded into a wide range of optoelectronic devices such as photodetectors, light‐emitting diodes, and lasers, demonstrating unprecedented improvements in device performance [4, 5, 6, 7].

Despite these advantages, precise control over the electronic energy level and its spatial distribution in OHPs remains a major challenge, significantly constraining their use in complex or integrated device architectures. In conventional semiconductor technologies, spatially defined electronic energy level modulation is typically realized through ion implantation or diffusion processes. However, such approaches are not readily transferable to halide perovskites due to their soft lattice, defect tolerance, and sensitivity to processing conditions. As a result, most reported modulation strategies in OHPs focus on modifying the global electronic properties of the material rather than enabling lateral, spatially programmable electronic energy level modulation.

A variety of modulation strategies have been explored (Table S1), including intrinsic or extrinsic defect engineering and charge transfer from adjacent layers or dopant species [8, 9]. While shallow defect engineering can modulate electronic energy levels by exploiting the defect‐tolerant nature of OHPs [10], conventional routes‐such as stoichiometric control via annealing or excess precursor addition—are governed by complex defect equilibria, limiting precise and reproducible control over defect type and density [11, 12, 13, 14]. Similarly, extrinsic dopants often interact strongly with native defects, leading to charge compensation or defect passivation rather than effective energy level modulation [15, 16, 17, 18, 19, 20, 21]. Interfacial charge transfer using selective contacts or molecular dopants has also been demonstrated [22, 23, 24, 25], but these approaches are largely confined to vertical device geometries and suffer from limited spatial resolution and uniformity [26].

Here, we report a fundamentally different energy level modulation paradigm that enables two‐dimensional, laterally defined surface‐potential junctions directly on the surface of widely used FAPbI_3_ perovskite thin films using electron‐beam (e‐beam) irradiation. Unlike conventional chemical or interfacial energy level modulation strategies, this approach relies solely on locally induced defect‐mediated electronic modulation, allowing continuous and spatially programmable tuning of the work function within a single perovskite layer. Kelvin probe force microscopy (KPFM) reveals that controlled e‐beam exposure leads to a linear increase in the local work function with micrometer‐scale spatial resolution. X‐ray photoelectron spectroscopy (XPS), together with density functional theory (DFT) calculations, elucidates that the observed work‐function modulation originates from e‐beam‐induced shallow defect states, predominantly associated with iodine‐related point defects. By selectively irradiating only part of the perovskite surface, we directly “write” a lateral surface‐potential‐defined junction without introducing extrinsic dopants or additional material interfaces. As a proof of concept, a perovskite photodetector incorporating an e‐beam‐defined lateral surface‐potential junction exhibits a microscopically resolved built‐in potential and pronounced rectifying behavior, demonstrating efficient and selective charge carrier separation. This e‐beam‐enabled, mask‐free strategy establishes a new route toward spatially programmable electronic architectures in halide perovskites, opening opportunities for planar optoelectronic devices and integrated functional patterns beyond the scope of conventional energy level modulation concepts.

## 2 Results and Discussion

**FIGURE 1:**
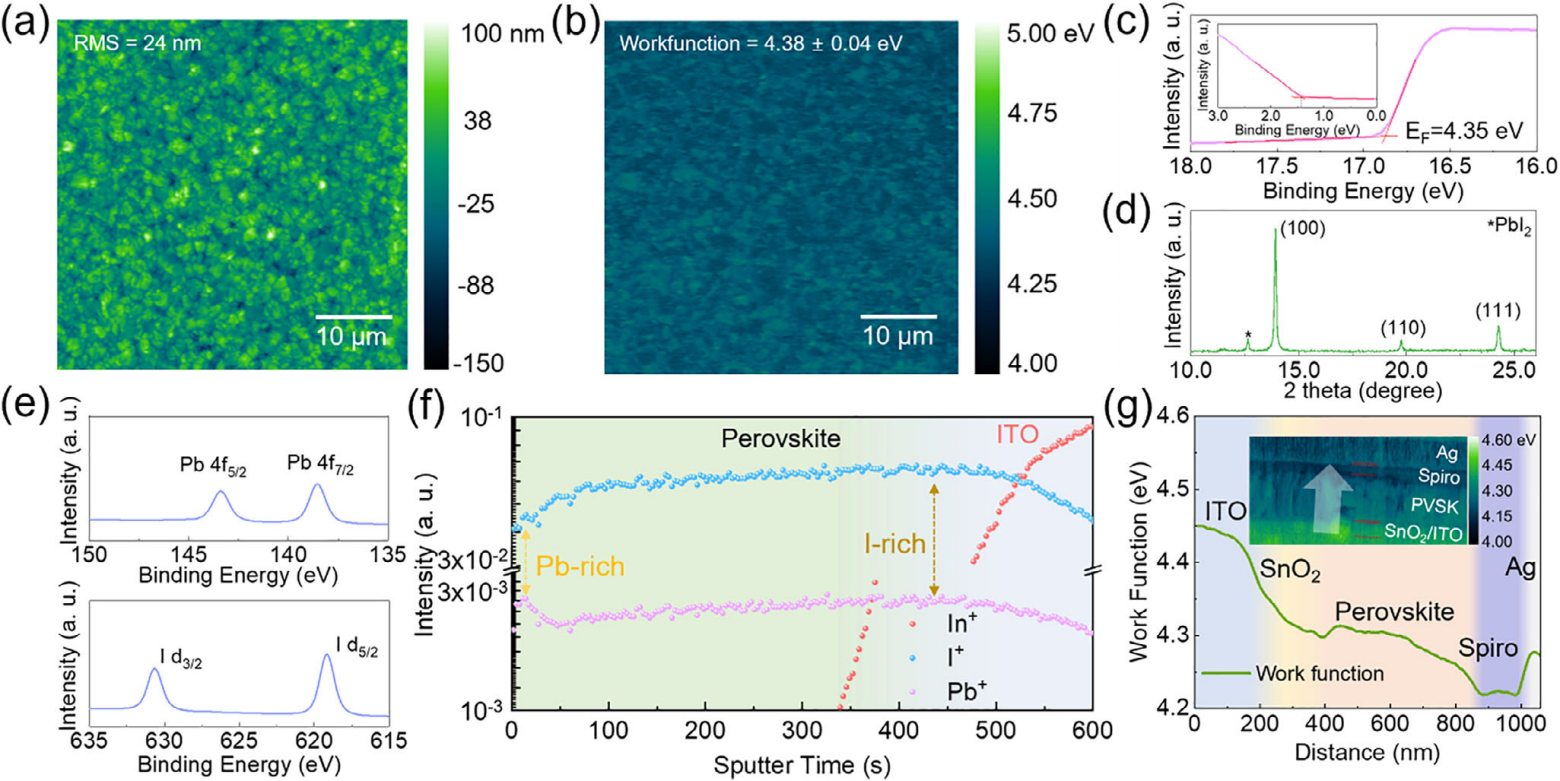
(a) Surface topography and (b) work function maps of the perovskite film obtained by Kelvin probe force microscopy (KPFM). (c) Ultraviolet photoelectron spectroscopy (UPS) spectrum and the extracted work function of the FAPbI_3_ perovskite thin film. (d) X‐ray diffraction pattern and (e) X‐ray photoelectron spectroscopy (XPS) spectrum of Pb 4f and I 3d core levels measured from the perovskite film. (f) Time‐of‐flight secondary ion mass spectrometry (ToF‐SIMS) depth profile of the perovskite film. (g) Cross‐sectional KPFM results showing a work function image and corresponding line profiles acquired along the white arrow.

We employed a widely used FAPbI_3_ composition incorporating methylammonium chloride (MACl) as a prototypical system [27]. As shown in Figure 1a,b, KPFM measurements revealed a uniform surface coverage (roughness ∼ 24 nm) with an average surface work function of 4.38 ± 0.04 eV, which is further supported by the work function value measured using ultraviolet photoelectron spectroscopy (UPS) (Figure 1c, 4.35 eV). In consideration of the valence band maximum (VBM, 4.24 eV) and conduction band minimum (CBM, 5.77 eV), the perovskite film fabricated in our study demonstrated *n*‐type characteristics. The overall *n*‐type character of the FAPbI_3_ film might be related to PbI_2_ formation during the thermal annealing process, as revealed by XRD patterns in Figure 1d [28, 29]. From the XPS spectrum measured in Figure 1e, the calculated I:Pb ratio of 2.68 reflected an iodine‐depleted surface, probably resulting from the volatilization of organic iodide during the thermal annealing process (Table S2), thereby leading to the observed *n*‐type surface characteristics [30]. From time‐of‐flight secondary ion mass spectrometry (ToF‐SIMS) measurement of the film, we noted that the top surface is in PbI_2_‐rich state relative to the bottom surface (Figure 1f). Notably, the work function value measured for the bottom surface of the exfoliated film demonstrated relatively less *n*‐type characteristics (4.70 ± 0.04 eV, Figure S1). We fabricated both the p–i–n and n–i–p structured perovskite solar cells (PSCs) with the architectures of ITO/Me‐4PACz/FAPbI_3_/C60/BCP/Ag and ITO/SnO_2_/FAPbI_3_/Spiro‐OMeTAD/Ag, respectively. The PSCs exhibited negligible hysteresis in the *J–V* curves, yielding a power conversion efficiency (PCE) of 25.5% for p–i–n and 22.1% for n–i–p (Figure S2 and Table S3). The stabilized PCE of 25.3% and 20.9% were achieved under continuous bias, supporting moderate quality of the tested FAPbI_3_ perovskite films. From cross‐sectional KPFM measurement in Figure 1g, the vertical work function gradient was observed in the neutral region (Figure 1g). In the vicinity of the top and bottom surfaces (outside the depletion region), the relatively *n*‐type top surface region (4.23 eV) compared to the bottom surface region (4.31 eV) indicated that there is a depth‐dependent work function distribution in the perovskite films. This indicated that stoichiometric distribution (and thus distribution of defects) can result in locally different work function distributions in the film. This motivated us to explore the possibility of modulating the distribution of defects to control the local work function in the perovskite films.

Direct e‐beam irradiation is commonly employed in electron microscopy measurements of halide perovskite thin films. Due to the fragile halide perovskite lattice, the high‐energy electron beam is subject to damaging the perovskite lattice unless delicately controlled [31]. Precisely controlled e‐beam irradiation can contribute to polishing of perovskite film surface, thereby eliminating defects and enhancing photoluminescence (PL) intensity [32, 33]. Based on these studies, we planned to explore the possibility of utilizing e‐beam irradiation to control the intrinsic defects in halide perovskite thin films.

**FIGURE 2:**
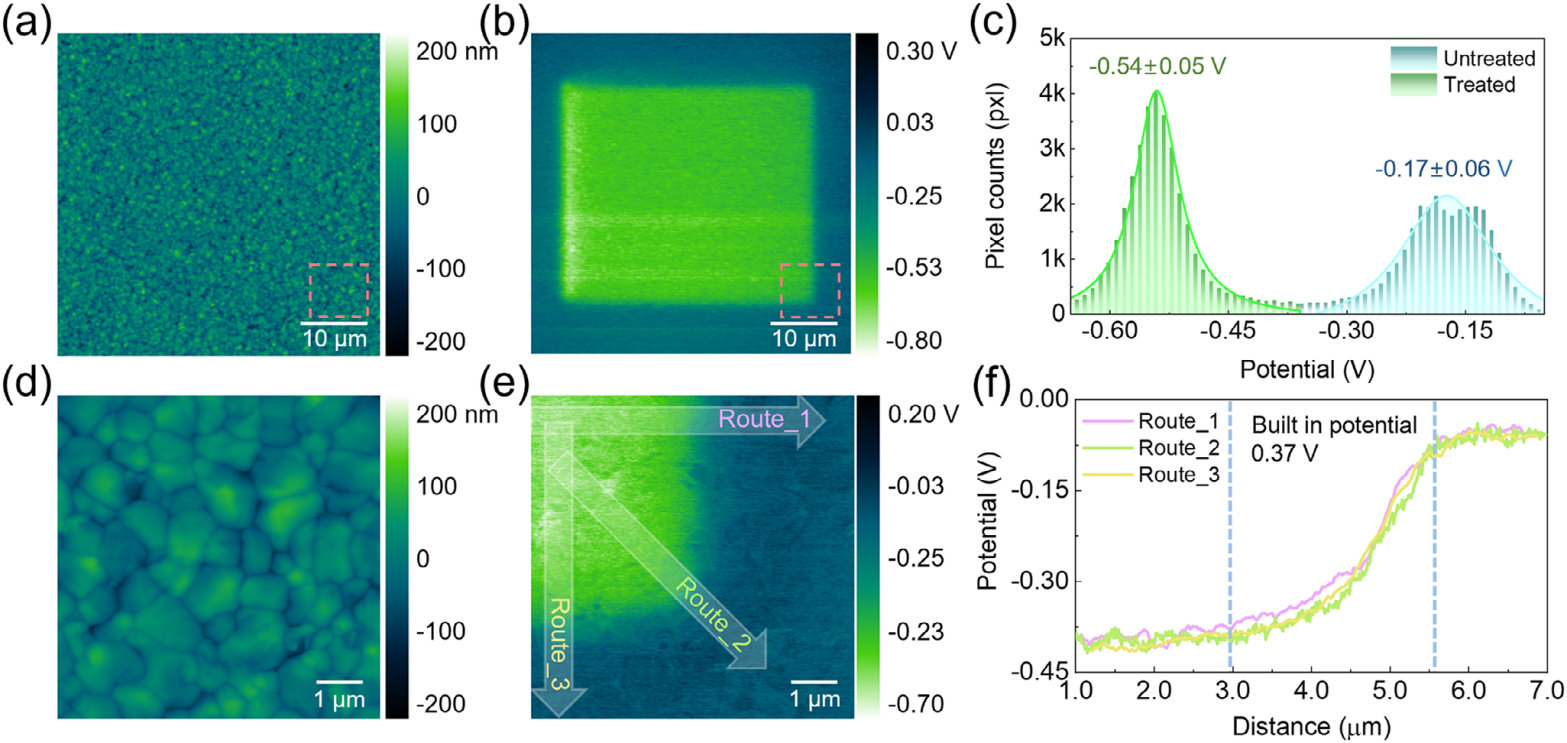
(a) Topography images and (b) corresponding contact potential difference (CPD) maps of the e‐beam‐treated samples (25 kV, 3 min), where CPD represents the relative surface potential with respect to the tip. (c) CPD distribution extracted from (b). (d,e) Magnified views of the regions marked by red dashed squares in (a,b), respectively. (f) Line profiles extracted along multiple white arrows in (e).

To investigate the influence of e‐beam exposure on the local morphology and surface potential of perovskite films, the KPFM measurement was performed to take advantage of its high spatial resolution and sensitivity to topographical and electrical features. Similar to the pristine perovskite films, the samples exposed to e‐beam irradiation with an acceleration voltage of 25 kV for 3 min (Figure 2a) exhibited a smooth surface with a roughness of ∼29 nm. While the untreated area displayed an average surface potential of −0.17 ± 0.06 V, the average surface potential of the e‐beam‐irradiated region shifted to −0.54 ± 0.05 V (a shift by ∼0.37 V), indicating an increase in work function (Figure 2b,c). The exposed area (40 µm  ×  40 µm) exhibited a clear contrast in surface potential and thus a consistent vacuum level shift of about −0.37 V. Figure 2d,e shows topographic and surface potential images measured at the boundary between the pristine and e‐beam irradiated region. Contrary to the clear change in surface potential, the magnified topographic images of the boundary region (Figure 2d) demonstrated no noticeable difference between the e‐beam‐treated and untreated regions. Multiple line profiles across the boundary region showed consistent stepwise potential shifts, indicating that a microscale lateral surface potential junction is formed with built‐in potential (Figure 2e,f).

To examine the stability of the e‐beam‐induced work‐function modulation, additional KPFM measurements were performed under dark‐storage and light‐exposure conditions. For the sample irradiated with a 25 kV e‐beam for 20 min, the same e‐beam‐dosed region retained its work‐function contrast over 14 days without noticeable topographic degradation (Figure S3). In addition, the sample was vertically illuminated through the glass substrate from the bottom using a 0.5 W‐class white LED for 1 h, and KPFM measurements performed under dark conditions before and after illumination showed no significant change in either topography or work‐function contrast (Figure S4). These results support the retention of the e‐beam‐induced work‐function modulation under the tested temporal and light‐exposure conditions. However, we note that the present stability evaluation is limited to these specific conditions and does not include elevated‐temperature or other harsh external stress conditions.

**FIGURE 3:**
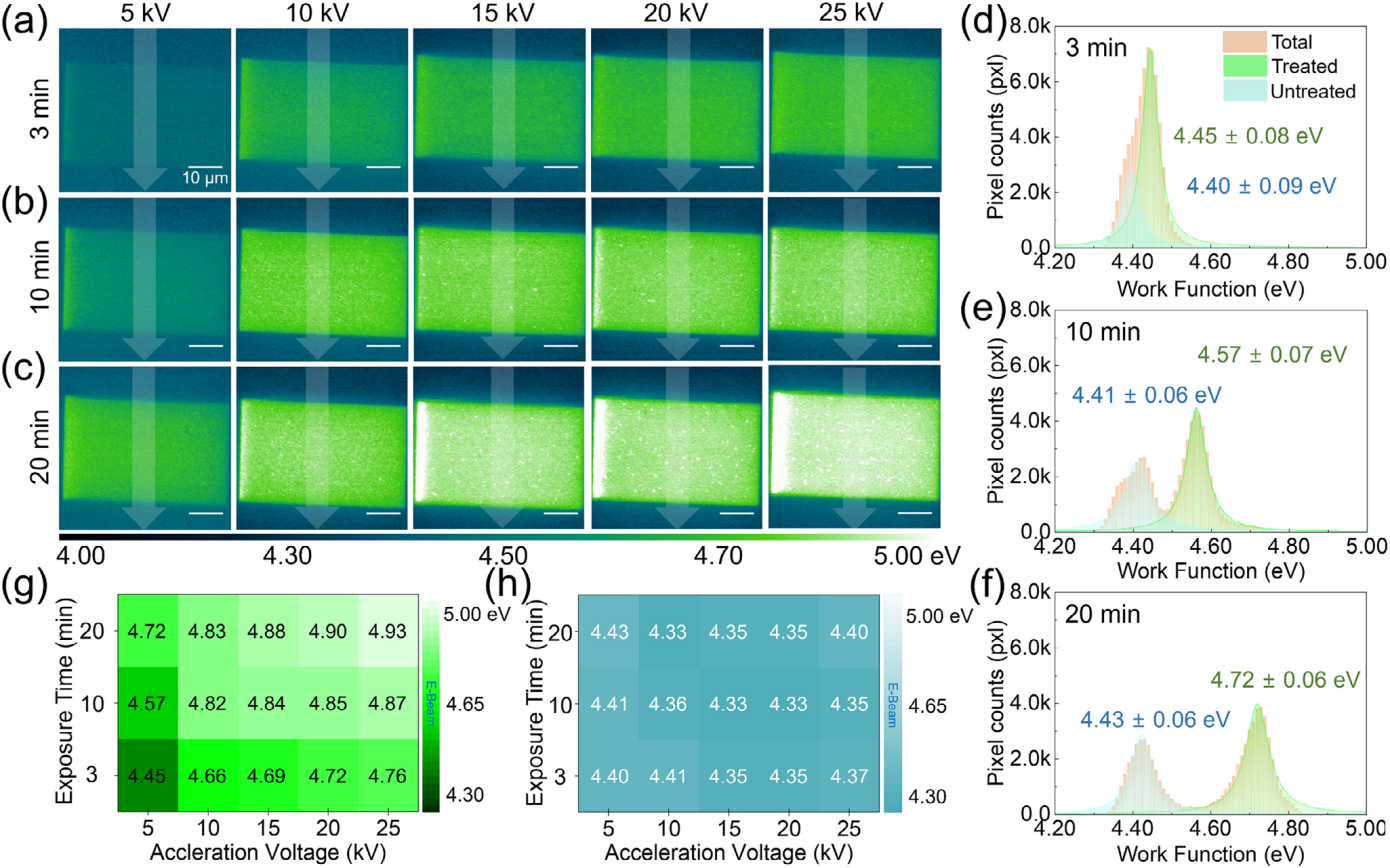
Work function maps acquired under varying e‐beam acceleration voltages with exposure times of (a) 3 min, (b) 10 min, and (c) 20 min. The area highlighted with a white arrow corresponds to the region used for extracting the work function line profile presented in Figure S9. Pixel distributions corresponding to the 5 kV‐dosed perovskite with exposure times of (d) 3 min, (e) 10 min, and (f) 20 min. Heat maps of the average surface work function of (g) e‐beam‐treated and (h) untreated areas.

To further investigate the relationship between e‐beam irradiation conditions and the resulting surface‐potential shift, the acceleration voltage and exposure duration were systematically varied across multiple samples and measurement regions, as shown in Figure 3a–c and Figures S5 and S6. Even with a minimal dose with an acceleration voltage of 5 kV for 3 min (Figure 3d), the exposed area demonstrated a upward shift in the average work function from its initial value of 4.35 ± 0.02 eV to 4.45 ± 0.08 eV. The longer exposure of 10 and 20 min formed a clear potential boundary between the treated and untreated region, where the average work function of the treated region increased to 4.57 ± 0.07 and 4.72 ± 0.06 eV, respectively (Figure 3e,f). The work function shift at a given exposure time become more pronounced as the acceleration voltage increased (Figure 3g). The irradiation of e‐beam with 25 kV induced a progressive work function shift from 4.35 ± 0.02 to 4.76 ± 0.07, 4.87 ± 0.05, and 4.93 ± 0.05 eV, after 3, 10, and 20 min of exposure, respectively (Table S4). The exposure‐time‐dependent trend reflects an increase in total electron dose at a given acceleration voltage, whereas the acceleration‐voltage‐dependent trend reflects the influence of incident electron energy rather than a simple increase in electron fluence. Detailed SEM irradiation parameters, calibration‐based beam‐current estimates, dose rates, and total electron doses are provided in Note S1. The pixelwise distribution of the work function further demonstrated a narrower distribution as the acceleration voltage increased (Figure S7). Meanwhile, the average work function of untreated areas remained almost unchanged (Figure 3h; Figure S7 and Table S5). The integrated line profiles for each condition were extracted along the white arrows in each image where the stepwise potential profiles are clearly visible at the boundary region (Figure S9). As e‐beam irradiation intensity increased with prolonged irradiation time or higher acceleration voltage, the built‐in electric field at the boundary region was correspondingly enhanced (Tables S6–S8).

**FIGURE 4:**
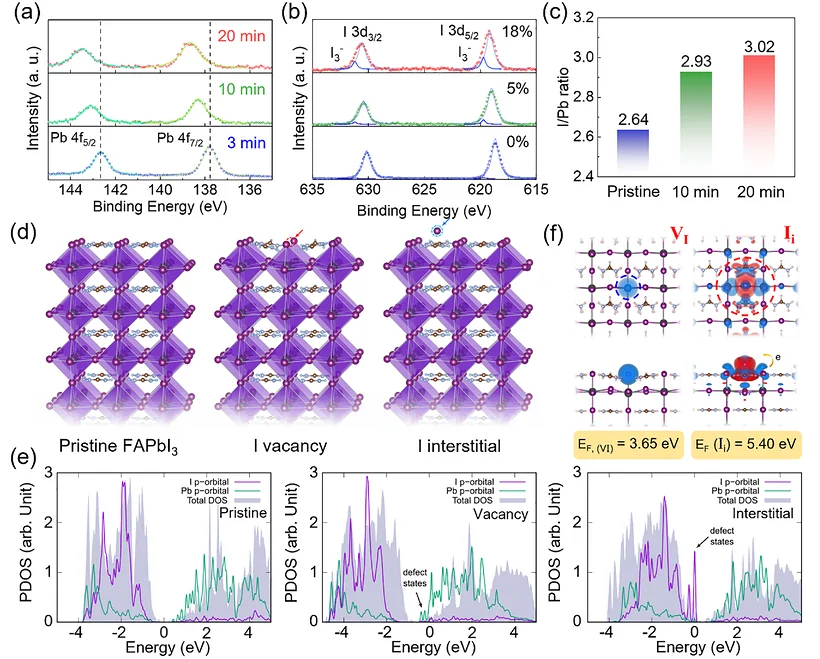
(a,b) XPS spectra of the Pb 4f and I 3d core levels and (c) the corresponding calculated I/Pb ratio under different exposure conditions. The solid lines represent the fitted curves obtained using a Lorentzian function. (d) Structural models of the FA‐I‐terminated FAPbI_3_ surface in three configurations: pristine, with an iodine vacancy (V_I_), and with a surface‐localized excess iodine defect (I_i_). The positions of the V_I_ and I_i_ defects are marked by red and blue arrows, respectively. (e) Projected density of states (PDOS) of Pb and I atoms near the defect sites for each configuration, along with the total DOS scaled for comparison. Defect‐induced in‐gap states are indicated by black arrows. (f) Differential charge density plots showing charge redistribution upon defect formation. Charge depletion is observed around the vacancy site (blue), while charge accumulation occurs near the surface excess‐iodine site (red), suggesting that the excess iodine atom draws electrons from the FAPbI_3_ surface.

To elucidate the underlying mechanisms for e‐beam‐induced work function change, X‐ray photoelectron spectroscopy (XPS) measurements were performed with varying irradiation times at an acceleration voltage of 5 kV. The C 1s peak at a binding energy of 285.0 eV, corresponding to C─C bonding, was used to calibrate all the spectra (Figure S10) [34]. For the pristine perovskite film, the Pb 4f peaks appeared at 137.8 eV, consistent with the peak from Pb^2+^ [35]. Upon e‐beam exposure, the Pb 4f peaks gradually shifted to higher binding energies, reaching 138.7 eV after 20 min of irradiation, with no noticeable emergence of a metallic Pb^0^ peak in lower binding‐energy region (Figure 4a). The shift of the Pb 4f peak toward higher binding energy implies a reduced electron density around the Pb‐I coordination sites [30, 36]. Simultaneously, the I 3d peaks also exhibited a gradual shift from 618.7 eV in the pristine film to 619.1 eV after 10 min and 619.4 eV after 20 min of e‐beam exposure (Figure 4b). Deconvolution of the I 3d peak unraveled the emergence of I_3_^−^ peak upon e‐beam irradiation, whose intensity increased with exposure time, reaching area ratio of 5% and 18% relative to the iodide peak after 10 and 20 min of irradiation, respectively [35, 37, 38]. The concurrent reduction in the total areas of both the I 3d peak, from 1.03 × 10^5^ to 3.38 × 10^4^, and the Pb 4f peak, from 3.09 × 10^4^ to 8.88 × 10^3^, upon irradiation implies that defect formation, rather than annihilation, dominates the process (Table S9).

To investigate the compositional changes in films with e‐beam irradiation, the I:Pb ratio was calculated from the XPS spectra as shown in Figure 4c. The pristine film showed an iodine‐poor surface with an I:Pb ratio of 2.64. Notably, the relative iodine content gradually increased upon e‐beam irradiation. The I:Pb ratio increased to 2.93 after 10 min of irradiation, and further increased to 3.02 after 20 min. The increase in iodine fraction with prolonged e‐beam irradiation correlated with the emergence of the I_3_^−^ peak in the XPS spectra, which is typically associated with interstitial‐iodine‐like defect or surface volatilization of I_2_ [35]. To further elucidate these changes in surface iodine‐related defect states, additional ToF‐SIMS depth profiling was performed on the e‐beam‐irradiated regions (Figure S11). Compared with the pristine film, the pronounced Pb^+^ signal near the top surface disappeared after e‐beam exposure and became uniformly distributed along the depth direction. Meanwhile, the surface‐depleted I^+^ signal observed in the pristine perovskite was recovered after e‐beam irradiation, with the surface I^+^ intensity becoming even exceeding that of the bulk region, indicating the formation of an iodine‐rich surface state. Given that the generation of iodine‐rich surface defects is favored at the surface region due to their low formation energy, the shallow states generated by interstitial‐iodine‐like defects might contribute to the work function shift upon e‐beam irradiation [39]. The confocal photoluminescence (PL) images of pristine and e‐beam‐irradiated films are shown in Figure S12. The e‐beam‐irradiated film exhibited slightly reduced local PL intensity relative to the pristine film, further substantiating the possible formation of interstitial‐iodine‐like shallow trap states induced by e‐beam exposure.

To complement experimental analyses, first‐principles DFT calculations were performed (Figure 4d–f). The FA‐I surface termination was adopted for the pristine FAPbI_3_ perovskite surface due to its thermodynamic stability [19, 40]. The effects of individual point defects were first investigated by introducing various possible defects (Figure S13). Among the possible defect states, an iodine vacancy (V_I_) and a surface‐localized excess iodine defect (I_i_) were mainly considered in a 2 × 2 slab model composed of six layers because of their relatively low formation energies compared with structurally distorting species and their close relevance to the observed work‐function shift (Figure 4d and Table S10). The V_I_ locally distorts the surrounding lattice structure, whereas the surface‐localized I, adsorbed as an adatom above the I─I bonded surface, induces minimal structural changes near the adsorption site. In Figure 4e, the projected density of states (PDOS) of Pb and I atoms adjacent to the defect sites are presented for each configuration. The V_I_ introduced in‐gap defect states just below the conduction band minimum (CBM), acting as donor levels to induce a decrease in work function. In contrast, the I_i_ generated shallow acceptor levels just above the valence band maximum (VBM), corresponding to an increase in work function. The distinct electronic contributions of the defects were clearly reflected in the calculated work function values of 4.61 eV for the pristine surface, 3.65 eV for the surface with V_I_, and 5.40 eV for the surface with I_i_ (Figure 4f). The charge density distribution plot showed electron accumulation near the I_i_, which is in agreement with the observed electron density reduction around the Pb^2+^ in XPS spectra. To simulate a more realistic scenario under e‐beam irradiation, we modeled I_i_‐V_I_ Frenkel defect pairs in which I_i_ is located near the surface and the V_I_ is positioned in the first, second, or third layer beneath the surface (Figure S14a). The calculated surface work function was found to increase as the V_I_ moved deeper into the bulk; 4.54 eV (first layer), 5.20 eV (second layer), and 5.32 eV (third layer). Total energy calculations revealed that the configuration with the I vacancy in the third layer is energetically most favorable, while those in the first and second layers are less stable by 48.9 and 34.9 meV per defect pair, respectively. Moreover, PDOS analysis (Figure S14b) shows that as the V_I_ migrates away from the surface, the associated defect states shift closer to the VBM, indicating a suppression of donor‐like behavior and a concurrent strengthening of acceptor‐like features. These results highlight that the experimentally observed increase in work function is predominantly governed by the presence of surface‐localized I interstitials, while the accompanying iodine vacancies preferentially diffuse into the bulk due to energetic stabilization. Once formed, the surface I_i_ is expected to remain localized over time, supported by the thermodynamic stability of the surface configuration. This interstitial–vacancy separation provides a physically plausible atomistic mechanism by which electron beam irradiation stabilizes the surface electronic states and contributes to the observed work function modulation in halide perovskite films.

**FIGURE 5:**
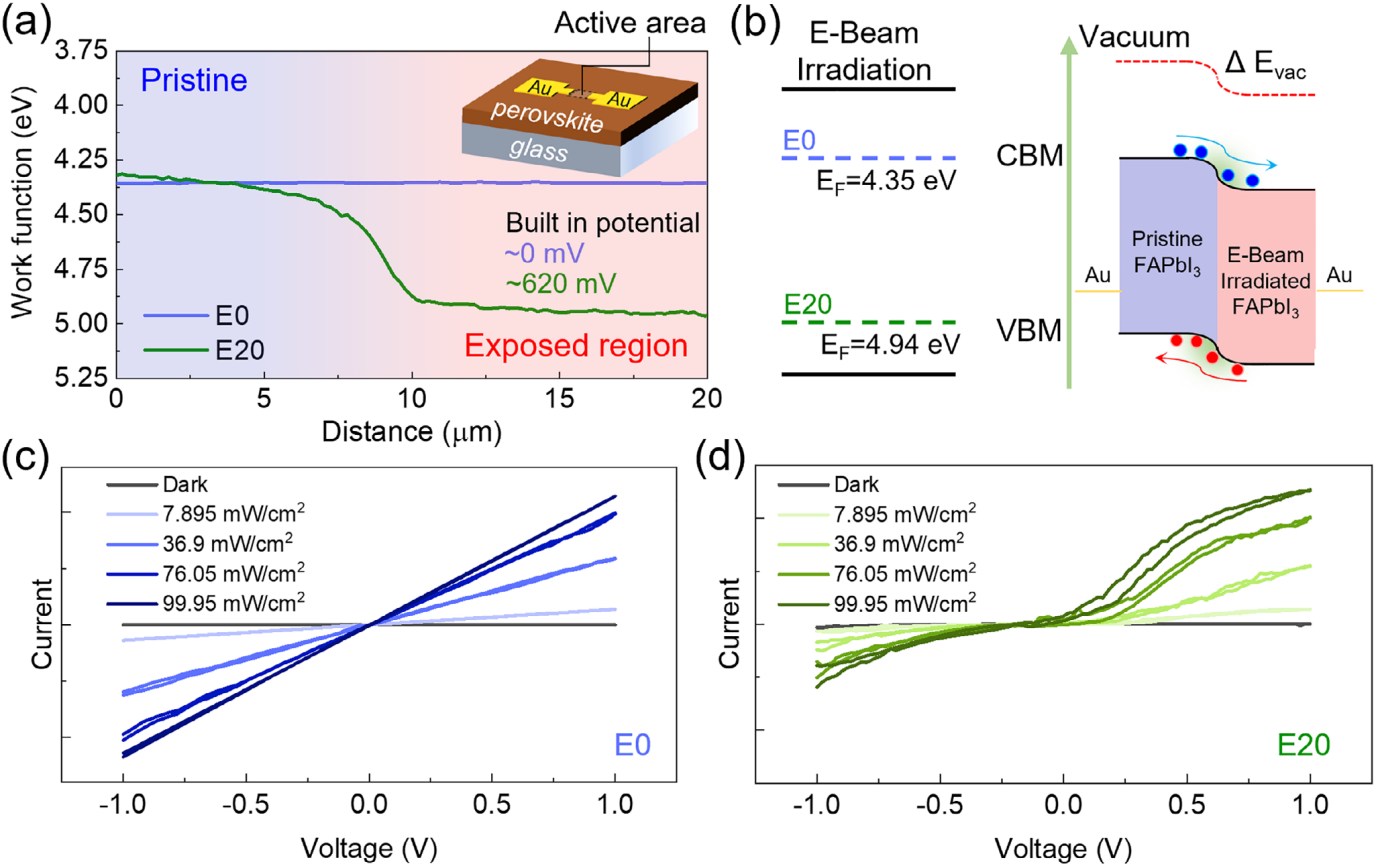
(a) Work function profile across the active area in the device illustration. (b) Schematic band diagrams of pristine and e‐beam‐exposed perovskite at an acceleration voltage of 25 kV for 20 min, along with the corresponding device band structure. (c,d) Photocurrent response of the E0 (no exposure) and E20 (25 kV, 20 min) devices as a function of illumination power density.

The e‐beam irradiation is capable of microscopic spatial control. To demonstrate proof‐of‐concept, PePD devices with a lateral Au/FAPbI_3_/Au configuration were adopted (Figure 5a). Half of the microscale active area (40 µm × 80 µm) within the device (80 µm × 80 µm) was selectively irradiated with a 25 kV electron beam for an exposure time of 40 min (E20), whereas the remaining half was left unexposed. Since the irradiated area in the device was twice as large as that used in the KPFM measurements, the exposure time was scaled proportionally to the irradiated area. Accordingly, the 40 µm × 80 µm region was irradiated for 40 min to maintain an areal dose equivalent to the 20 min condition confirmed by KPFM measurements; this device is denoted as E20. In contrast, the E0 device was entirely unirradiated. As shown in Figure 5a,b, e‐beam irradiation induced work function modulation and established a built‐in potential at the boundary between the exposed and intact perovskite surfaces. A lateral built‐in potential of approximately 620 mV was formed in the E20 device, whereas no discernible potential difference was observed in the E0 device. The photoresponse of the PePD under varying illumination intensities was evaluated in Figure 5c,d. The E0 device exhibited a mirror‐like inversion symmetry in its linear photocurrent response, whereas the E20 device displayed an asymmetric profile characterized by suppressed current under reverse bias and an exponential increase under forward bias. We further confirmed that *I–V* asymmetry was reproducibly observed in the half‐area‐irradiated devices across multiple independently fabricated devices (Figure S15). Furthermore, light‐intensity‐dependent I–V curves were measured for devices with different e‐beam‐irradiation areas, including non‐irradiated, half‐area‐irradiated, and full‐area‐irradiated devices (Figure S16 and Table S11). The rectifying behavior was observed only in the half‐area‐irradiated device, which showed a substantially higher rectification ratio of 3.201. By contrast, the non‐irradiated and full‐area‐irradiated devices exhibited nearly linear I–V characteristics, with rectification ratios of 1.017 and 0.891, respectively. However, the aging behavior of the device‐level rectification should be interpreted separately from the stability of the e‐beam‐induced work‐function modulation observed by surface KPFM (Figure S17). Although the KPFM results showed that the work‐function modulation remained stable for over 14 days without noticeable topographic degradation, the rectifying behavior of the devices weakened after 7 days of storage under the same conditions. This result implies that the degradation of rectification is not solely determined by the stability of the e‐beam‐induced potential gradient, but may also be strongly affected by device fabrication processes and device‐architecture‐related factors. These factors will be carefully considered in future studies. In particular, aging‐ and bias‐induced degradation at the metal/perovskite interface could modify the effective contact properties and consequently reduce the rectifying behavior of the lateral device [41, 42].

Overall, these results indicate that the built‐in potential formed between the irradiated and non‐irradiated regions governs the transport of photogenerated carriers, as its direction enables efficient current flow along a preferred pathway. This transition in rectification behavior arises from the local work function modulation, which enables more efficient charge‐carrier separation upon the establishment of an appropriate built‐in potential.

## 3 Conclusion

In this study, we demonstrated a rational strategy for spatial and continuous tuning of the work function in most commonly used FAPbI_3_ perovskite films by direct e‐beam irradiation. Surface KPFM analysis confirmed that the e‐beam‐irradiated regions exhibited a modulated work function compared to the pristine FAPbI_3_. This modulation was achieved through variations in both acceleration voltage and exposure duration, with higher voltages and longer irradiation time enabling a more pronounced vacuum level shift. The underlying modulation mechanism was elucidated through XPS analysis, which revealed a progressive shift of the Pb 4f peak toward higher binding energies with increasing e‐beam exposure, manifesting reduced electron density near the Pb‐I framework. The gradual emergence of a triiodide‐related peak associated with interstitial‐iodine‐like defect formation was attributed to the origin of the observed work function modulation by e‐beam irradiation. The theoretical calculations further supported that the interstitial‐iodine‐like defect from a Frenkel defect pair tends to be stabilized at the surface, contributing to shallow states and an increase in the work function. The proof‐of‐concept PePD was demonstrated, in which the e‐beam engineered lateral surface‐potential‐defined junction enables rectification behavior under light illumination. This work provides a promising foundation for defect‐mediated local work function modulation strategies for advanced optoelectronic devices based on FAPbI_3_ perovskite.

## Author Contributions

**Hyeonho Park**: methodology, investigation, formal analysis, validation, writing – review and editing. **Seung‐Gu Choi**: methodology, investigation, formal analysis, validation, writing – original draft, writing – review and editing. **Jae‐Hwan Kim**: investigation. **Dong‐Jun Lee**: investigation. **Sung‐Kwang Jung**: investigation. **Gwanghee Lee**: investigation. **HongSeok Kim**: investigation. **Kyung‐Hwan Jin**: investigation, conceptualization, writing – review and editing, supervision. **Jin‐Wook Lee**: conceptualization, investigation, writing – review and editing, funding acquisition, writing – original draft, project administration, supervision. **Seong Heon Kim**: supervision, conceptualization, investigation, funding acquisition, writing – original draft, writing – review and editing, project administration.

## Conflicts of Interest

The authors declare no conflicts of interest.

## Supporting information




**Supporting File**: advs77723‐sup‐0001‐SuppMat.pdf.

## Data Availability

All data needed to evaluate the conclusions in the paper are present in the paper or the supplementary materials.
