# Supplementary material for: Electron‐Beam Enables Spatially Resolved Defect‐Mediated Work Function Modulation in FAPbI3 Perovskite Thin Films
Source: Adv Sci (Weinh). 2026 Sep 16:e77723. Online ahead of print. doi: 10.1002/advs.77723 (PMC13583094; doi:10.1002/advs.77723)
Supplement: Supplementary file 1 [file ADVS-9999-e77723-s001.pdf]

Supporting Information

Electron-Beam Enables Spatially Resolved Defect-Mediated Work Function Modulation in $FAPbI_3$ Perovskite Thin Films

Hyeonho Park†, Seung-Gu Choi†, Jae-Hwan Kim†, Dong-Jun Lee, Sung-Kwang Jung, Gwanghee Lee, HongSeok Kim, Kyung-Hwan Jin, Jin-Wook Lee*, Seong Heon Kim**

†These authors contributed equally to this work.

Materials

Unless otherwise specified, all chemicals were purchased from Sigma-Aldrich. Formamidinium iodide (FAI, purity 99.99%), Methylammonium chloride (MACl, purity 99.99%) and Propane-1,3-diammonium iodide ($PDAI_2$) were obtained from Greatcell Solar. Lead iodide (PbI_2, purity 99.99%) and 4-(3,6-Dimethyl-9H-carbazol-9-yl)butylphosphonic Acid (Me-4PACz) were purchased from Tokyo Chemical Industry. C60 (purity, 99.5%) and tin oxide colloidal solution were purchased from OSM and Alfa Aesar, respectively. Spiro-OMeTAD was purchased from Luminescence technology corp.

Photo detector fabrication

Indium tin oxide (ITO) glass substrates were cleaned using an ultrasonicator with detergent, deionized water, acetone, and ethanol for 20 min, sequentially. The cleaned substrates were dried in an oven at 65 °C for 10 min. O_2 plasma treatment was applied to the ITO glass surface at a process pressure of ~ 10^2 Torr for 10 min. The perovskite precursor solution was prepared by dissolving 172.0 mg of FAI, 461.0 mg of PbI_2, and 15.2 mg of MACl in a mixed solvent (Dimethylformamide : *N*-methylpyrrolidone , 4:1 volume ratio) to obtain a final concentration of 2 M. 30 μL of perovskite solution was spin coated at 4000 rpm for 20 s, with 150 μL of diethyl ether (DE) dropped 13 s before completion. As-spun films were annealed at 100 °C for 5 min, followed by 150 °C for 30 min. 60 nm-thick top Au electrode with a lateral configuration was deposited through thermal evaporation at deposition rate of 0.5 Å s^{-1} under a vacuum level of ~10^{-7} Torr.

Solar cell fabrication

p-i-n structured devices: ITO glass substrates were patterned by chemical etching using HCl, followed by sequential sonication in detergent, deionized water, acetone, and ethanol for 20 min each. 100 W of Oxygen plasma treatment was applied for 10 min, after which the substrates were transferred into a N_2 filled glovebox. 200 μL of Me-4PACz in ethanol (0.3 mg mL^{-1}) was spin-coated at 3000 rpm for 30 s, followed by thermal annealing at 100 °C for 10 min. 60 μL of perovskite precursor solution was spin-coated at 1000 rpm for 10 s and 5000 rpm for 20 s. During the second step, 200 μL of chlorobenzene was dropped onto the film 5 s before the end of spin-coating process. The as-spun films were subsequently annealed at 100 °C for 60 min. 50 μL of $PDAI_2$ (0.2 mg mL^{-1} in isopropanol) was spin-coated at 4000 rpm for 45 s, followed by annealing at 100 °C for 5 min for passivation. Finally, 30 nm of C60 (0.2 Å s^{-1}), 8 nm of BCP (0.2 Å s^{-1}), and 100 nm of Ag (0.5 Å s^{-1}) were thermally evaporated in sequence to form the electron transport layer (ETL) and top electrode. The aperture size was defined as 0.05 cm^2 using a shadow mask.

n-i-p structured devices: Patterned fluorine-doped tin oxide (FTO) glass substrates were sequentially cleaned in an ultrasonic bath using detergent, acetone, deionized water and ethanol for 20 min each. The cleaned substrates were treated with ultraviolet ozone for 60 min. 0.25 mL of diluted SnO_2 colloidal solution (3.0 wt%) was spread onto the cleaned FTO glass and spin-coated at 3000 rpm for 30 s, followed by annealing at 150 °C for 30 min. The annealed SnO_2 films

were treated with UVO for 30 min prior to perovskite deposition. The perovskite precursor solution was spin-coated at 4000 rpm for 20 s, with 150 μL of DE dropped after 7 s, followed by sequential annealing at 100 °C for 5 min and then at 150 °C for 30 min. A spiro-OMeTAD solution was dropped onto the spinning perovskite-coated substrate after 5 s and spun for an additional 20 s. Finally, an 80 nm thick silver was thermally evaporated at a deposition rate of 0.5 Å s^{-1}. The aperture size was defined as 0.125 cm^2 using a shadow mask.

Material and Device Characterization

All PePD measurements were conducted in a vacuum chamber probe station system (~10^{-2} Torr) equipped with an optical fiber-guided halogen illuminator (FOK-100W, Fiber Optic Korea) and a Keithley 4200 SCS source meter. Confocal photoluminescence (PL) measurements with a spatial resolution below 200 nm were performed using a laser scanning microscopy system (NanoBase) equipped with a 532 nm laser as the excitation source. The mapping was conducted by scanning areas of 50 × 50 μm^2 on each detector sample. Current density-voltage (J-V) curves were obtained using a Keithley 2400 source meter under AM 1.5G one-sun (100 mW cm^{-2}) illumination generated by a solar simulator (Oriel sol3A, class AAA) equipped with a 450 W xenon lamp (Newport 6280NS). The light intensity was adjusted using an NREL-calibrated Si solar cell with a KG-5 filter. The *J-V* curves were measured at a scan rate of 130 mV s^{-1}. UPS spectra were obtained by photoelectron spectrometer system (NEXSA, ThermoFisher Scientific). High-resolution X-ray photoelectron spectroscopy was conducted using a commercial XPS instrument (AXIS Supra+, Kratos Analytical Ltd.) with a monochromatic Al Kα X-ray source. The OrbiSIMS instrument used in this study was an M6 hybrid SIMS (IONTOF GmbH, Münster, Germany) installed at the Yeongnam Regional Center of the Korea Basic Science Institute (KBSI, instrument code: BS102). The main chamber pressure of the instrument was maintained at $1.3 \times 10^{-6} \sim 1.1 \times 10^{-9}$ mbar. For in-depth analysis, a 10 keV, Ar_{1200} gas cluster (a current of 2nA) was used with a cycle time of 200 μs. Secondary ion masses were measured in positive mode over an area of 50 μm^2 (random raster mode) with 128 × 128 pixel resolution during the data acquisition for each sample using a 30 keV, Bi_3^+ primary ion beam, with a crater size of 200μm^2. X-ray diffraction (XRD) pattern was measured by a Rigaku Smart Lab SE diffractometer with Cu-Kα radiation (λ = 1.5406 Å). The scan rate was set to 4°/min, with a step size of 0.02°. Kelvin probe force microscopy measurements were performed using a commercial AFM system integrated in Ar-filled glove box (NX-10, Park Systems). The Pt-coated probe was adopted to maximize the spatial resolution of work function images by considering high electrical sensitivity. To accurately calculate the work function from the measured contact potential difference (CPD) representing the relative surface potential compared to the tip, the conductive cantilever was calibrated before and after each measurement. This calibration was performed using the offset determined by measuring the work function of highly ordered pyrolytic graphite (HOPG), which has a constant value of 4.65 eV.

Computational Methods

First-principles calculations based on density functional theory (DFT) were performed using the projector augmented wave (PAW) method as implemented in the Vienna Ab Initio Simulation Package (VASP).[1-2] The Perdew–Burke–Ernzerhof (PBE) form of the generalized gradient approximation (GGA) was employed for the exchange–correlation functional. For the bulk and thin-film electronic structure of $FAPbI_3$, the experimental lattice parameters were adopted.[3] Surface slab models were constructed along the [001] direction using a five-unit-cell-thick slab, with a vacuum region of approximately 20 Å to avoid spurious interactions between periodic images. The FA-I-terminated surface was considered, and point defects including iodine vacancies (V_I) and iodine interstitials (I_i) were introduced within a 2 × 2 supercell.

All atomic positions were fully relaxed until the residual forces were less than 0.01 eV/Å. The slab calculations were performed using a plane-wave energy cutoff of 500 eV and a 7 × 7 × 1 Monkhorst–Pack *k*-point mesh. Spin–orbit coupling (SOC) was included self-consistently in all electronic structure calculations.

Supplementary Note 1: **E-beam irradiation and dose quantification**

E-beam irradiation was performed using a tungsten-filament SEM operated in full-field raster scan mode. The beam was continuously raster-scanned over a predefined rectangular field rather than being held at a stationary point. For the KPFM irradiation series, the exposed area was approximately 40×40 μm² at ×5000 magnification. For the E20 photodetector device, the exposed region was approximately 40 μm×80 μm at ×2500 magnification, corresponding to two adjacent 40 μm×40 μm unit areas. To maintain the same area-normalized electron dose as the 25 kV, 20 min KPFM irradiation condition, the total raster exposure time for the 40 μm×80 μm E20 device region was set to 40 min. Therefore, the E20 device condition represents a 20 min-equivalent exposure per 40 μm×40 μm unit area, not a 20 min total exposure over the full 40 μm×80 μm device region. The $FAPbI_3$ film edge was electrically connected to the SEM stage using conductive carbon tape to provide a charge dissipation pathway during irradiation.

The common SEM-column settings used for the irradiation experiments were a 30 μm aperture, tungsten filament electron source, spot-size setting of 12, and working distance of 11.1 mm. The spot-size value of 12 is an instrument-defined SEM setting rather than an independently measured probe diameter. The acceleration voltage and emission current were set to 5 kV/61 μA, 10 kV/58 μA, 15 kV/55 μA, 20 kV/52 μA, and 25 kV/50 μA.

The beam current was estimated from an experimentally measured reference SEM-column condition. Under 15 kV acceleration voltage, 55 μA emission current, 30 μm aperture, tungsten filament source, spot-size setting of 12, working distance of 15–16 mm, and ×300 magnification, the beam current was measured to be 50–60 pA. Because beam current was not independently measured at every historical irradiation condition, the beam current at each acceleration voltage was estimated by scaling the measured 15 kV reference beam-current range according to the emission current used during irradiation:

$$I_{b,est}(V) = I_{b,ref} \times \frac{I_{em}(V)}{I_{em,ref}}$$

where $I_{b,ref}$ is the measured beam current range of 50–60 pA under the reference condition, $I_{em,ref}$ is the reference emission current of 55 μA, and $I_{em}(V)$ is the emission current used at each acceleration voltage. The resulting values are reported as calibration-based estimated beam currents.

The total electron dose was calculated as:

$$D_{total} = \frac{I_{b,est}t}{eA}$$

and the dose rate was calculated as:

$$D_{rate} = \frac{I_{b,est}}{eA}$$

where $I_{b,est}$, t, e, and A are the estimated beam current, total raster exposure time, elementary charge, and raster-scanned irradiation area, respectively. This definition follows the standard electron microscopy treatment of electron dose or fluence as the number of incident electrons divided by the irradiated area. The electron dose is reported in $e^{-}Å^{-2}$, using the conversion factor $1e^{-}Å^{-2} = 10^{16}e^{-}cm^{-2}$. The pixel-level dwell time was not independently controlled or recorded for each historical irradiation run because the exposure was performed by continuous full-field raster scanning. Therefore, the scan mode, exposed field area, total raster exposure time, spot-size setting, estimated beam current, dose rate, and total electron dose are reported as the reproducibility parameters.

It should be noted that the acceleration voltage determines the incident electron energy, whereas the electron dose is determined by beam current, irradiated area, and exposure time. Because the emission current was slightly reduced at higher acceleration voltages, the estimated electron dose does not increase with acceleration voltage at a fixed exposure time. Therefore, the acceleration-voltage-dependent work function modulation should be interpreted as an incident-electron-energy-dependent effect, while the exposure-time-dependent modulation corresponds to an increase in total electron dose.

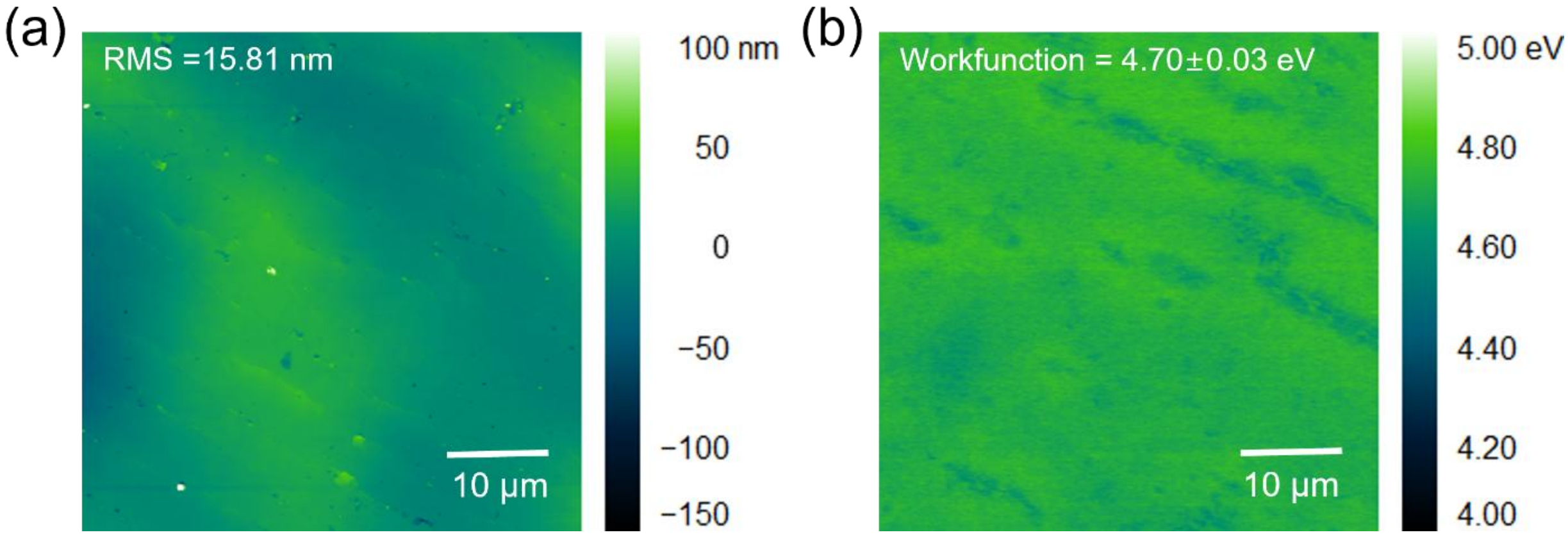

Figure S1. (a) Topography and (b) workfunction maps of perovskite bottom surface.

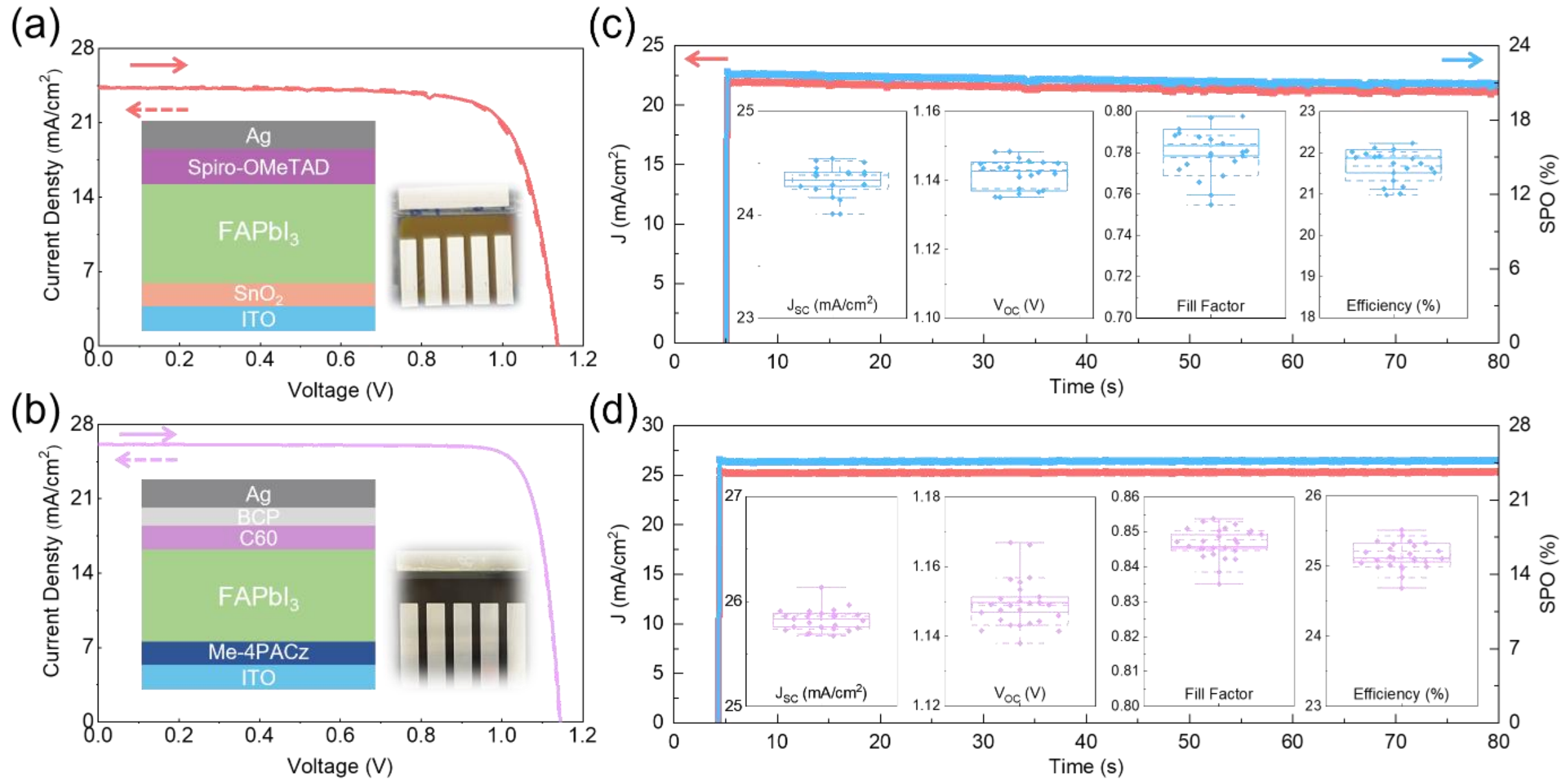

Figure S2. Representative J-V curves of perovskite solar cells (PSCs) with (a) conventional n-i-p architecture (ITO/SnO_2/$FAPbI_3$/Spiro-OMeTAD/Ag) and (b) inverted p-i-n architecture (ITO/Me-4PACz/$FAPbI_3$/C60/BCP/Ag). Stabilized power output measurements and the inset statistical distributions of the photovoltaic parameters, short-circuit current density (J_{SC}), open-circuit voltage (V_{OC}), fill factor (FF), and power conversion efficiency (PCE), for the (c) n-i-p and (d) p-i-n devices.

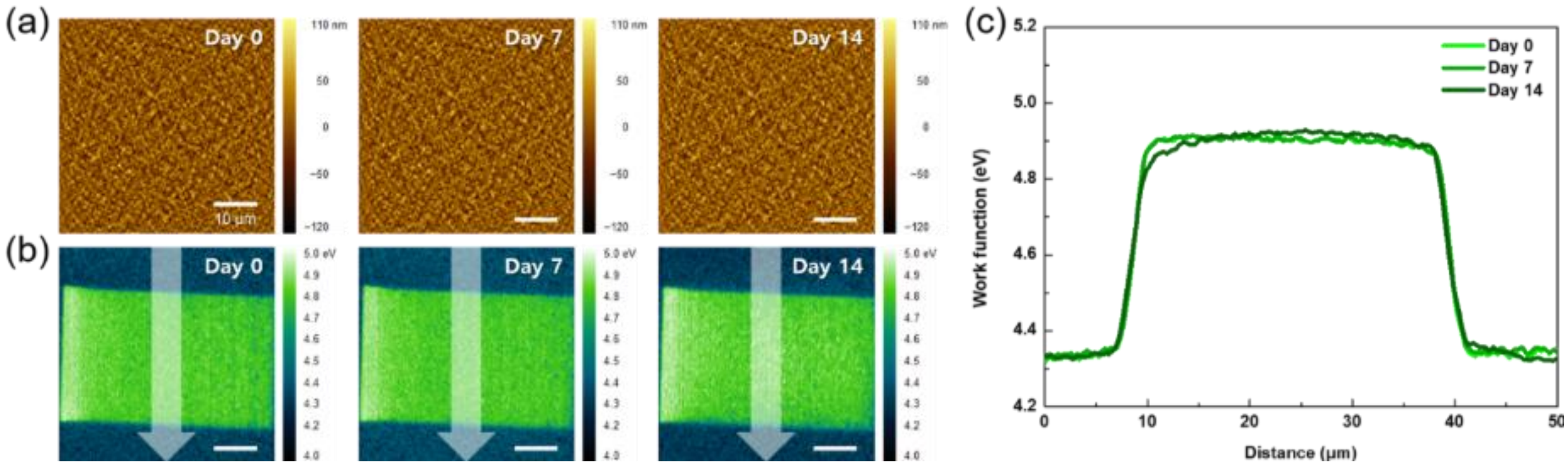

Figure S3. (a) Topography maps and (b) corresponding work-function maps measured at the same location on the same $FAPbI_3$ sample on Day 0, Day 7, and Day 14. (c) Averaged line profiles extracted from the work-function maps in (b).

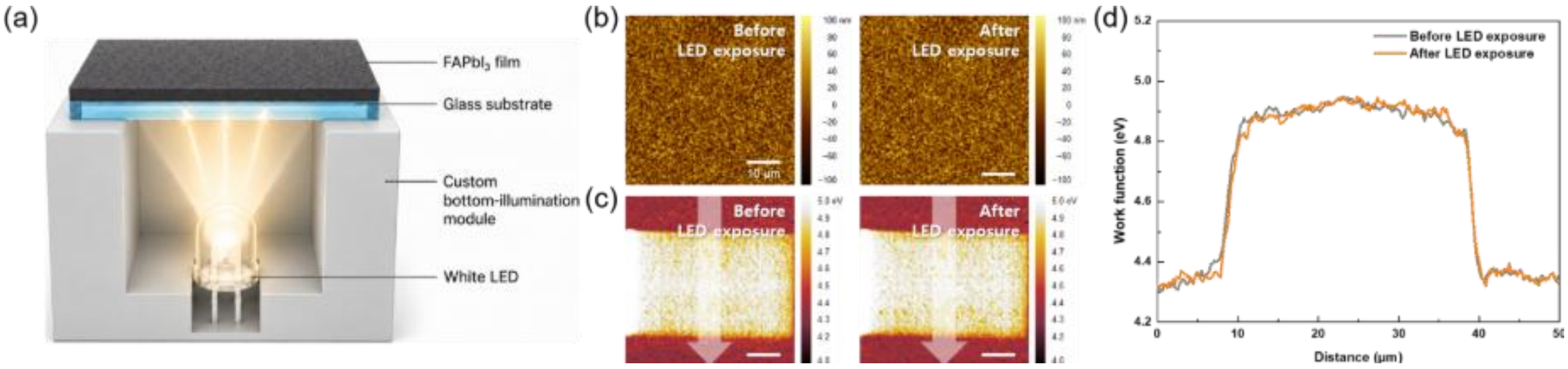

Figure S4. (a) Schematic illustration of white LED illuminating the bottom of a sample vertically using a custom-built optical module. (b) Topography maps and (c) corresponding work-function maps measured at the same location on the same $FAPbI_3$ sample on before and after LED Exposure (d) Averaged line profiles extracted from the work-function maps in (c).

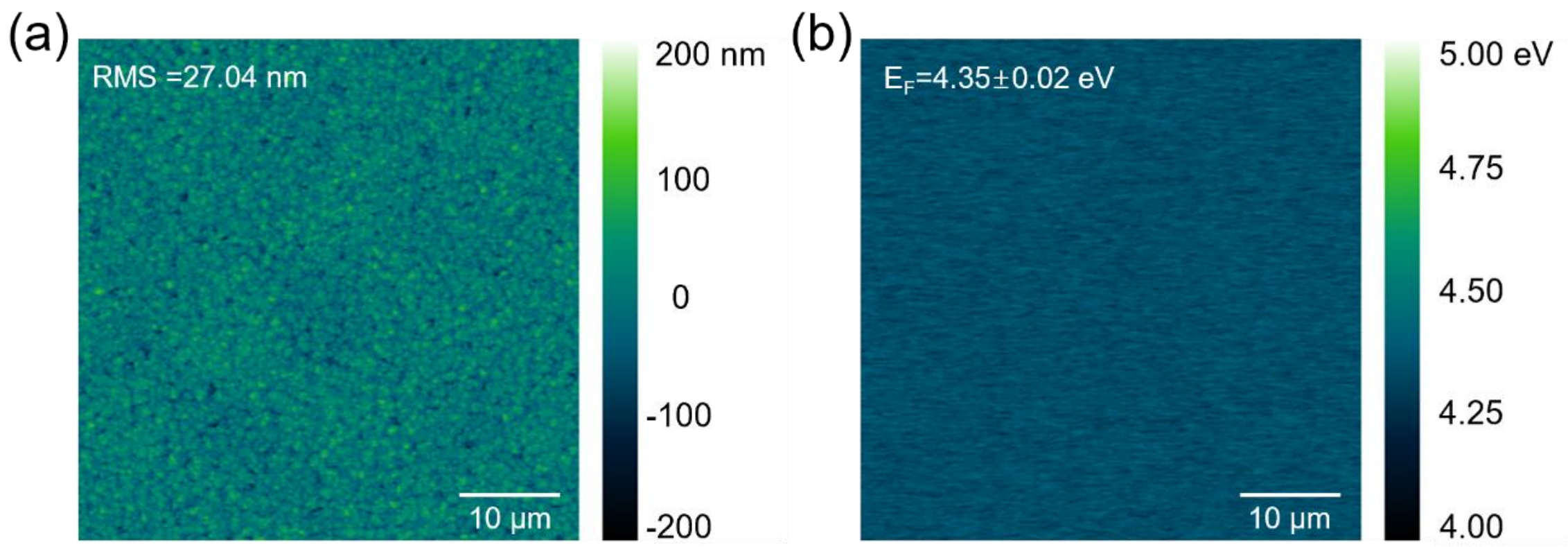

Figure S5. (a) Topography and (b) work function maps of top surface of perovskite film prior to e-beam exposure.

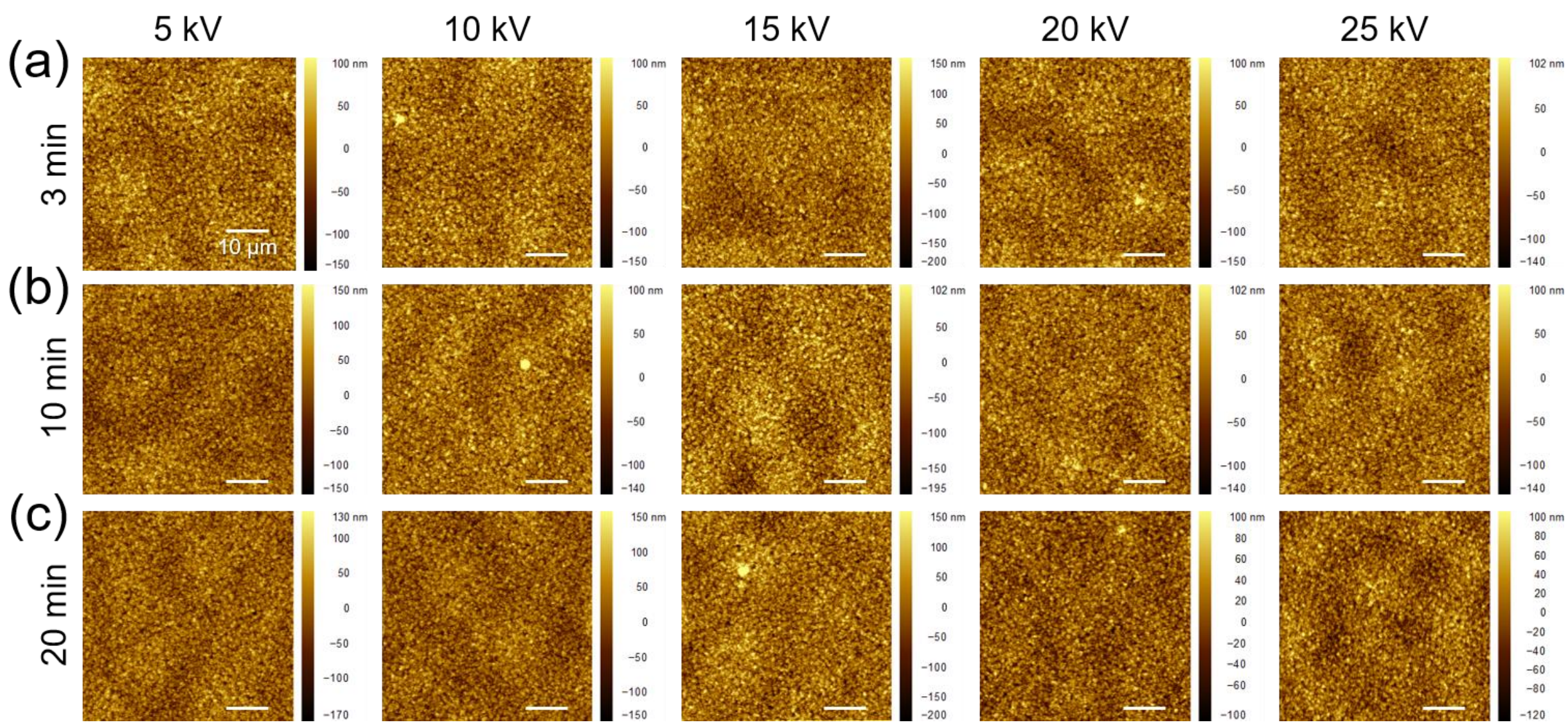

Figure S6. Topographic maps corresponding to independent KPFM datasets. (a–c) Z height images acquired simultaneously with the KPFM work-function maps shown in **Figure 3** under e-beam exposure times of (a) 3 min, (b) 10 min, and (c) 20 min, respectively, with acceleration voltages ranging from 5 to 25 kV.

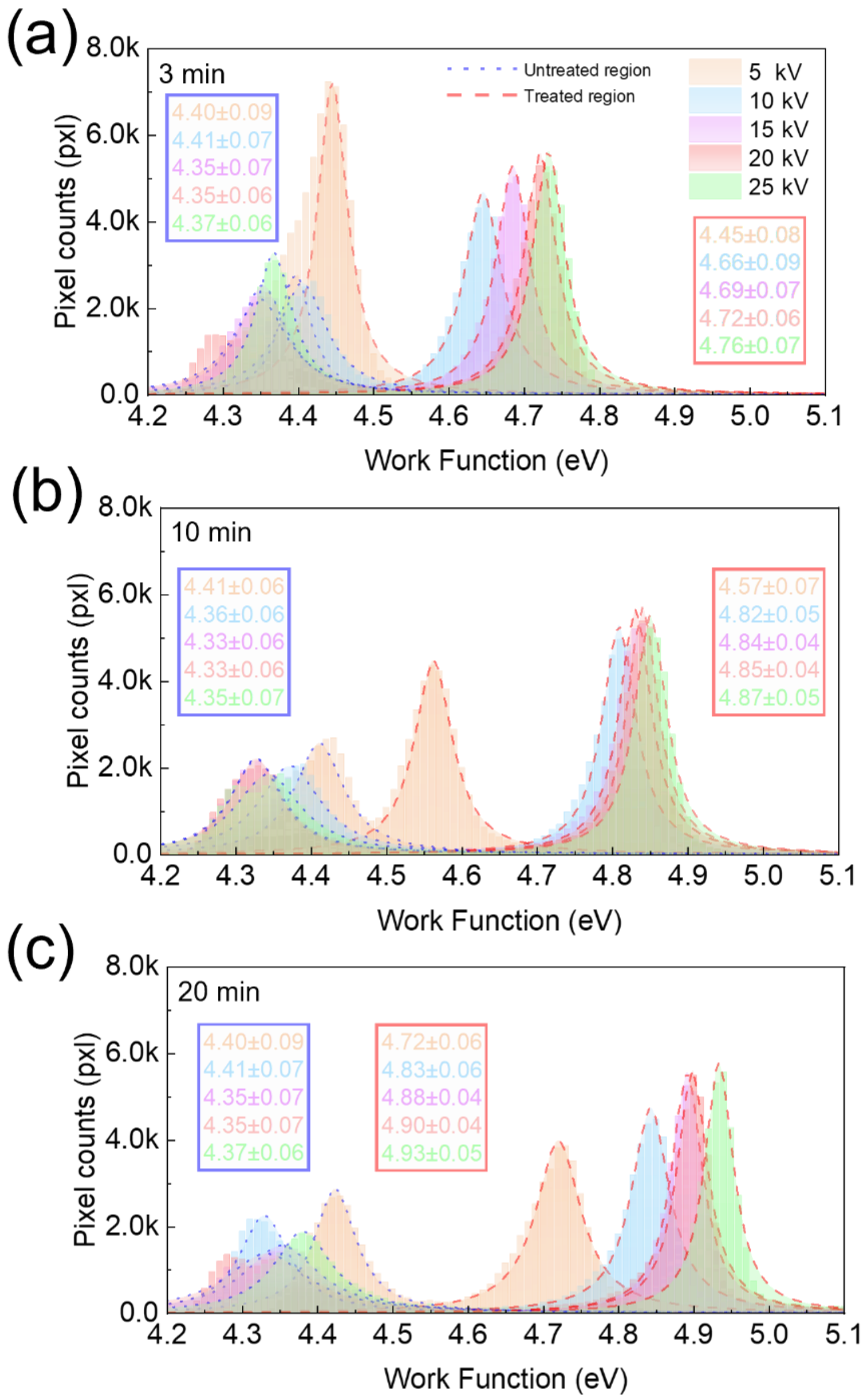

Figure S7. Workfunction pixel distribution under varying e-beam acceleration voltages with exposure times of (a) 3 min, (b) 10 min, and (c) 20 min.

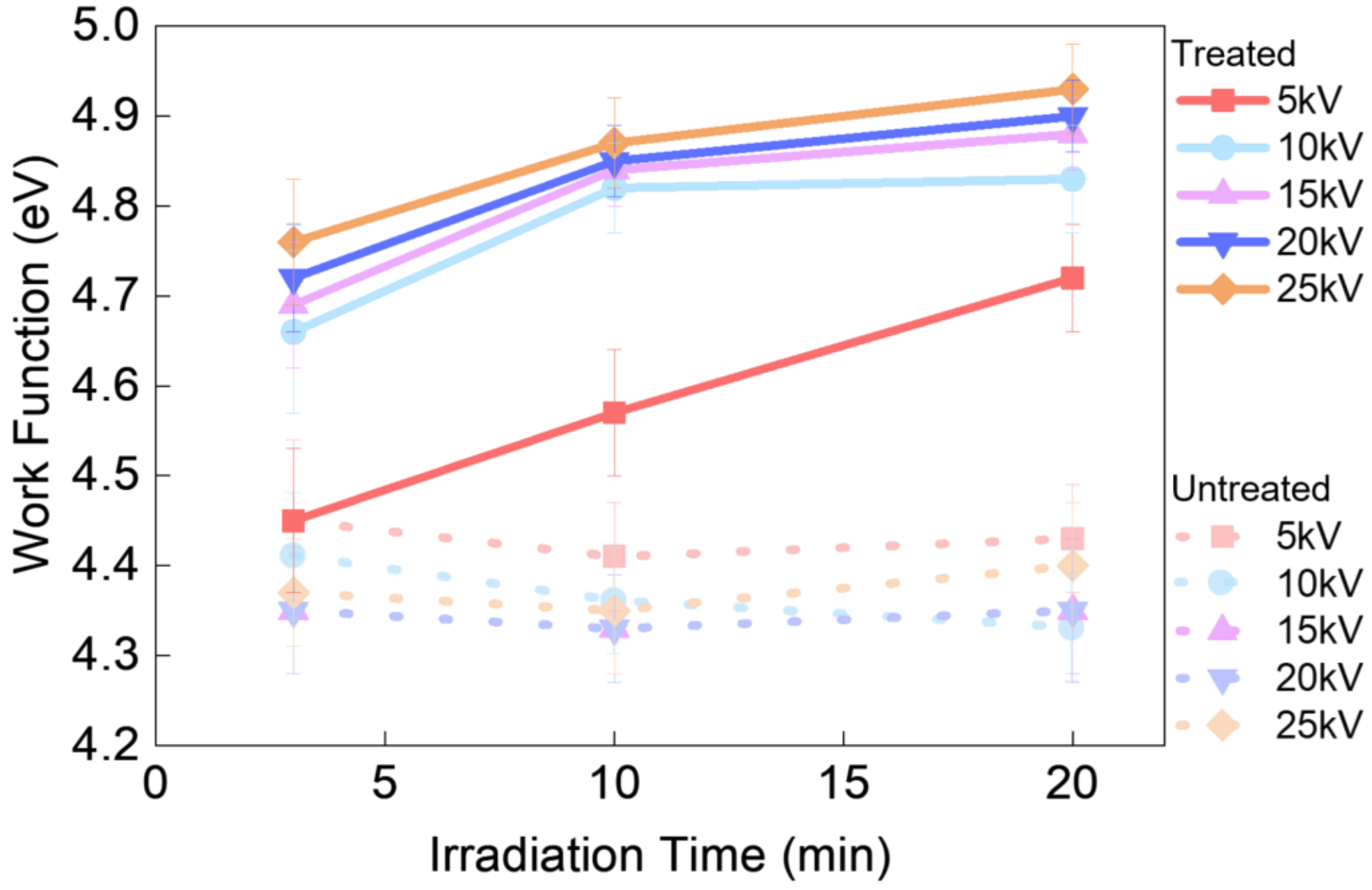

Figure S8. Average work function changes in treated and untreated regions of the e-beam irradiated samples as a function of acceleration voltage and exposure time.

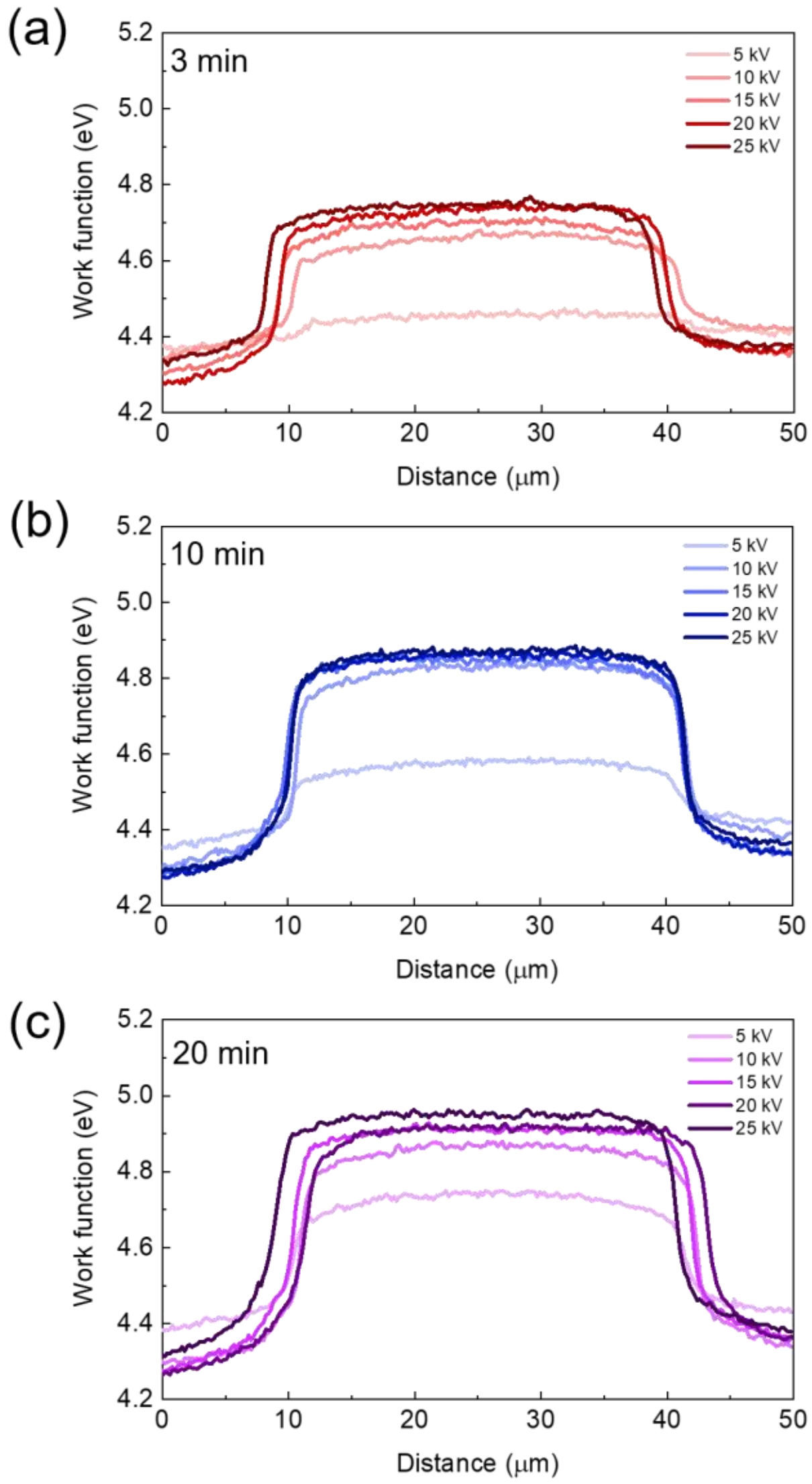

Figure S9. Integrated line profiles under varying acceleration voltages were extracted along the white arrows for exposure time of (a) 3 min, (b) 10 min, and (c) 20 min.

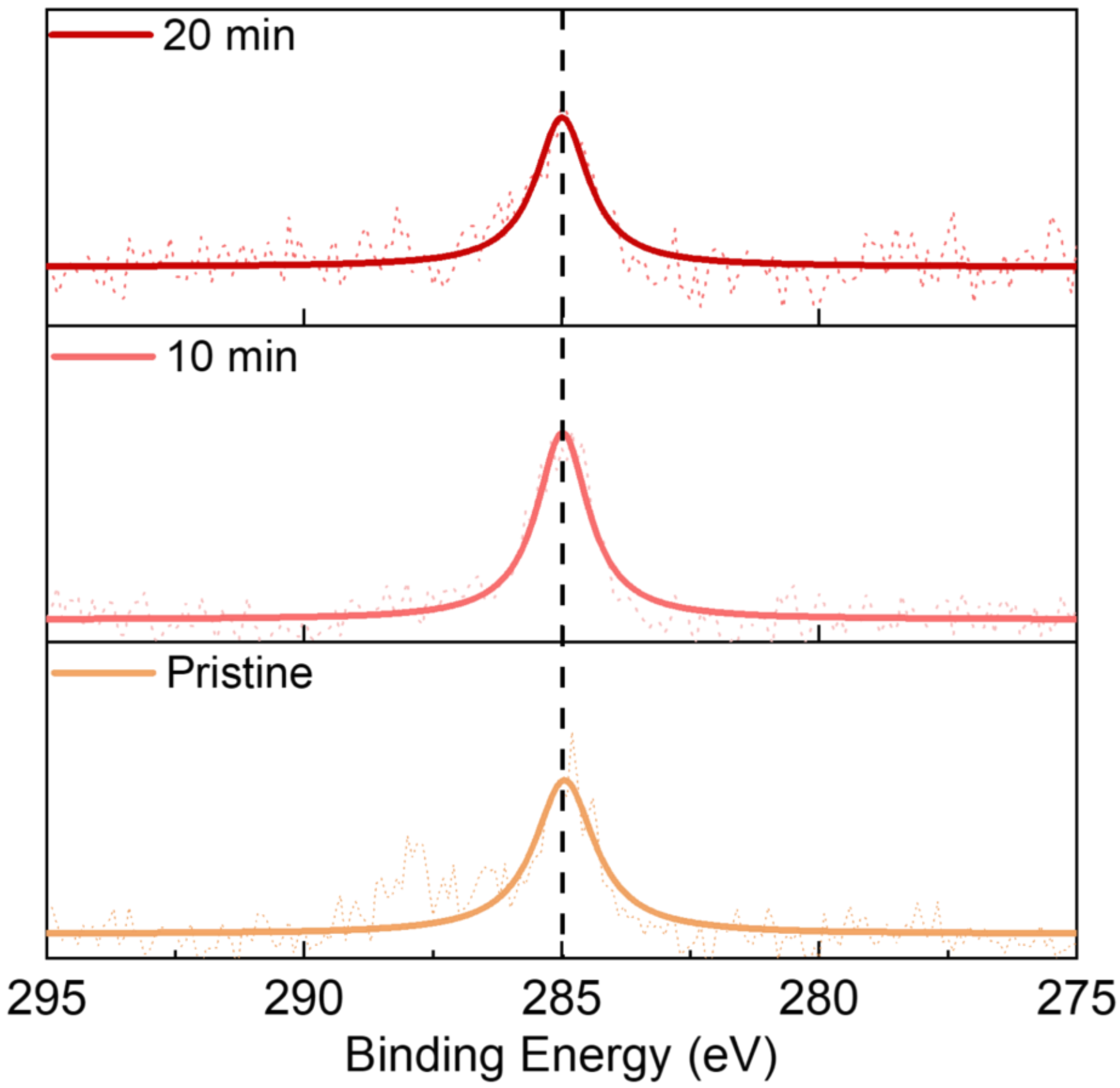

Figure S10. XPS spectra of the C 1s core levels for pristine, 10 min, and 20 min dosed films under 5 kV e-beam irradiation.

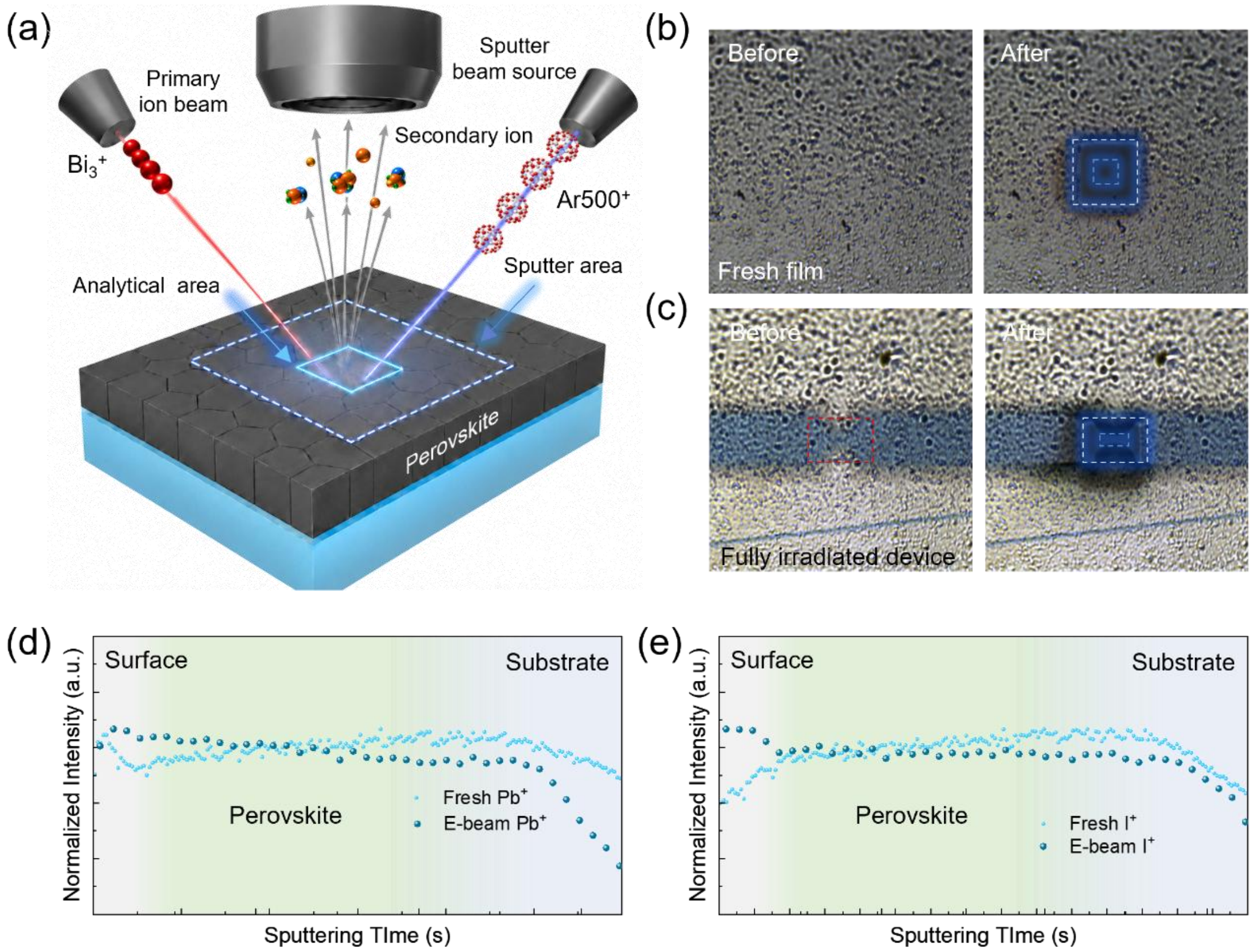

Figure S11. (a) Schematic illustration of time-of-flight secondary ion mass spectrometry (ToF-SIMS) performed using a dual-beam configuration with a gas cluster ion beam sputtering source. The cyan square and bright blue dashed line indicate the region of interest (ROI) for analysis and the sputtered area, respectively. Photographs of (b) the pristine perovskite film and (c) the fully e-beam-irradiated perovskite photodetector before and after depth profiling. Ion depth profiles of (d) Pb^+ and (e) I^+ in the pristine film and e-beam-irradiated region.

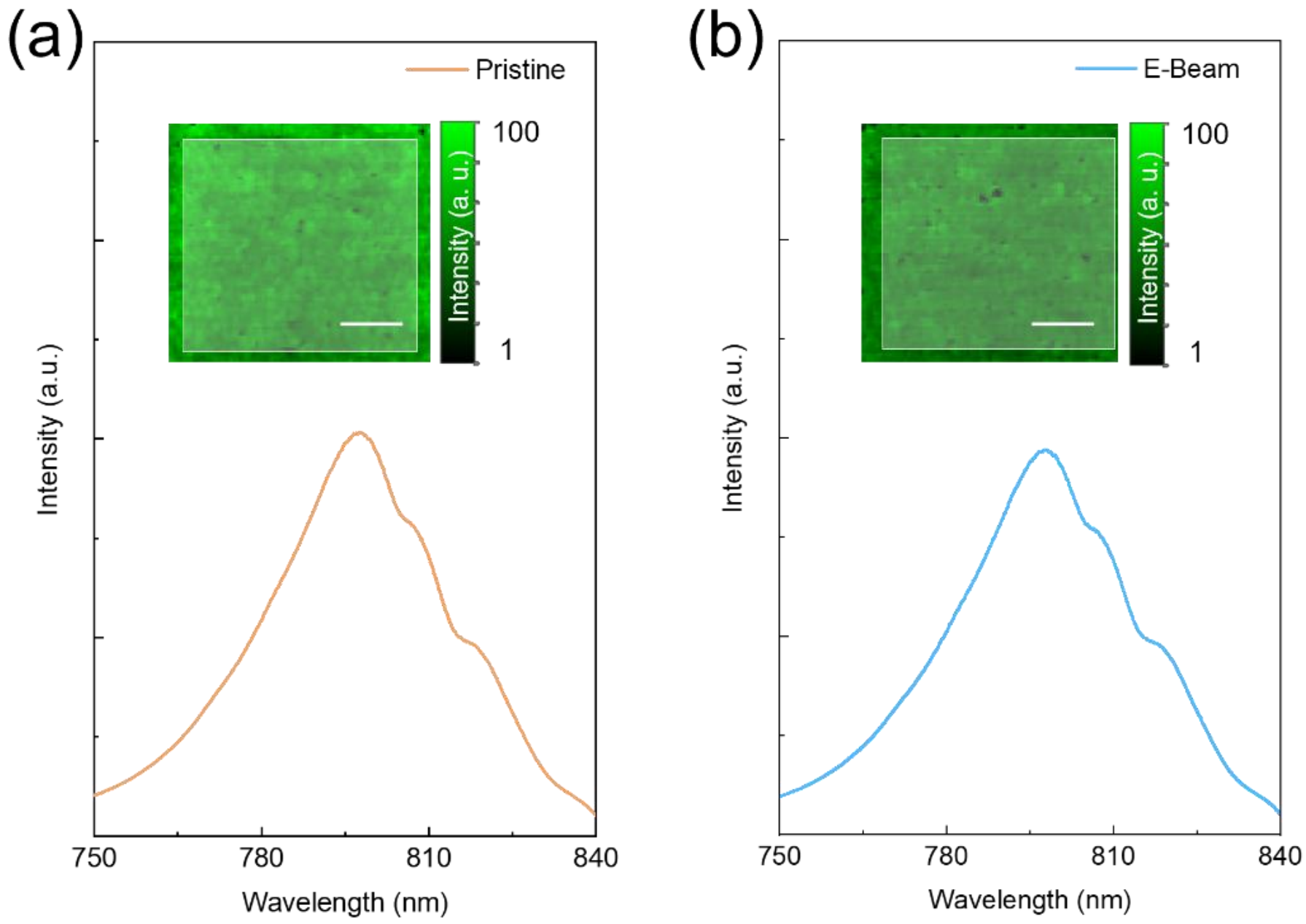

Figure S12. Photoluminescence mapping images of (a) pristine and (b) e-beam treated perovskite films. The corresponding intensity spectra were extracted from the white marked regions.

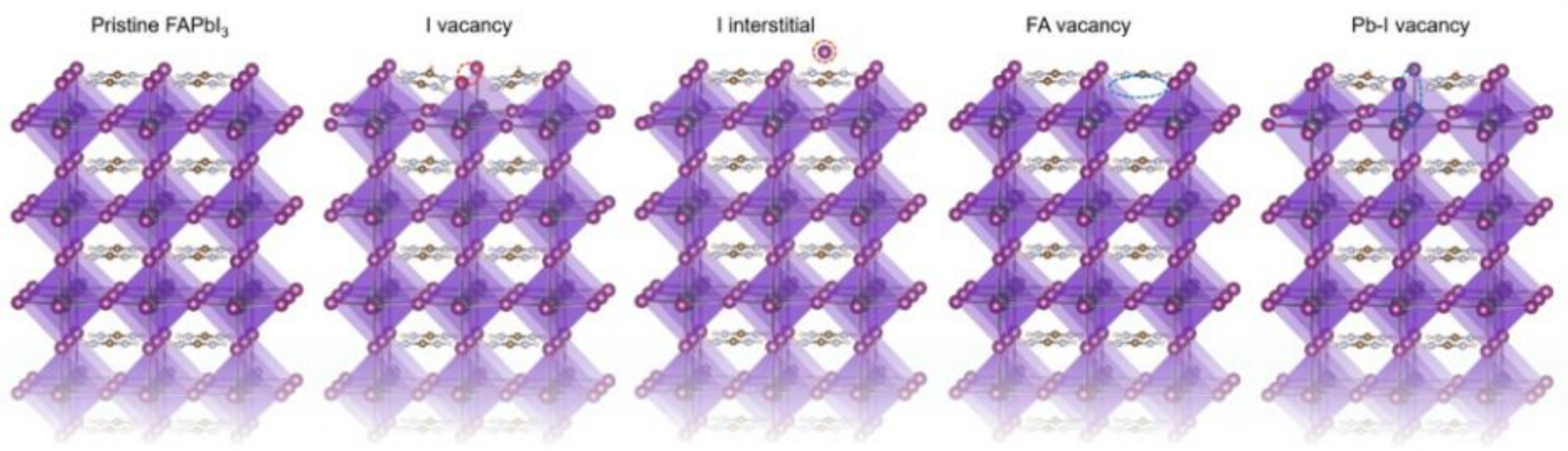

Figure S13. Structural models of pristine and defective $FAPbI_3$ surfaces used in the DFT calculations. The optimized slab structures of the pristine $FAPbI_3$ surface and representative defect configurations are shown, including an iodine vacancy (V_I), iodine interstitial (I_i), formamidinium vacancy (V_{FA}), and Pb–I vacancy-pair defect ($V_{Pb–I}$). The defect sites are highlighted with dashed circles. These models were used to evaluate how different beam-induced defects modify the surface electronic structure and work function of $FAPbI_3$.

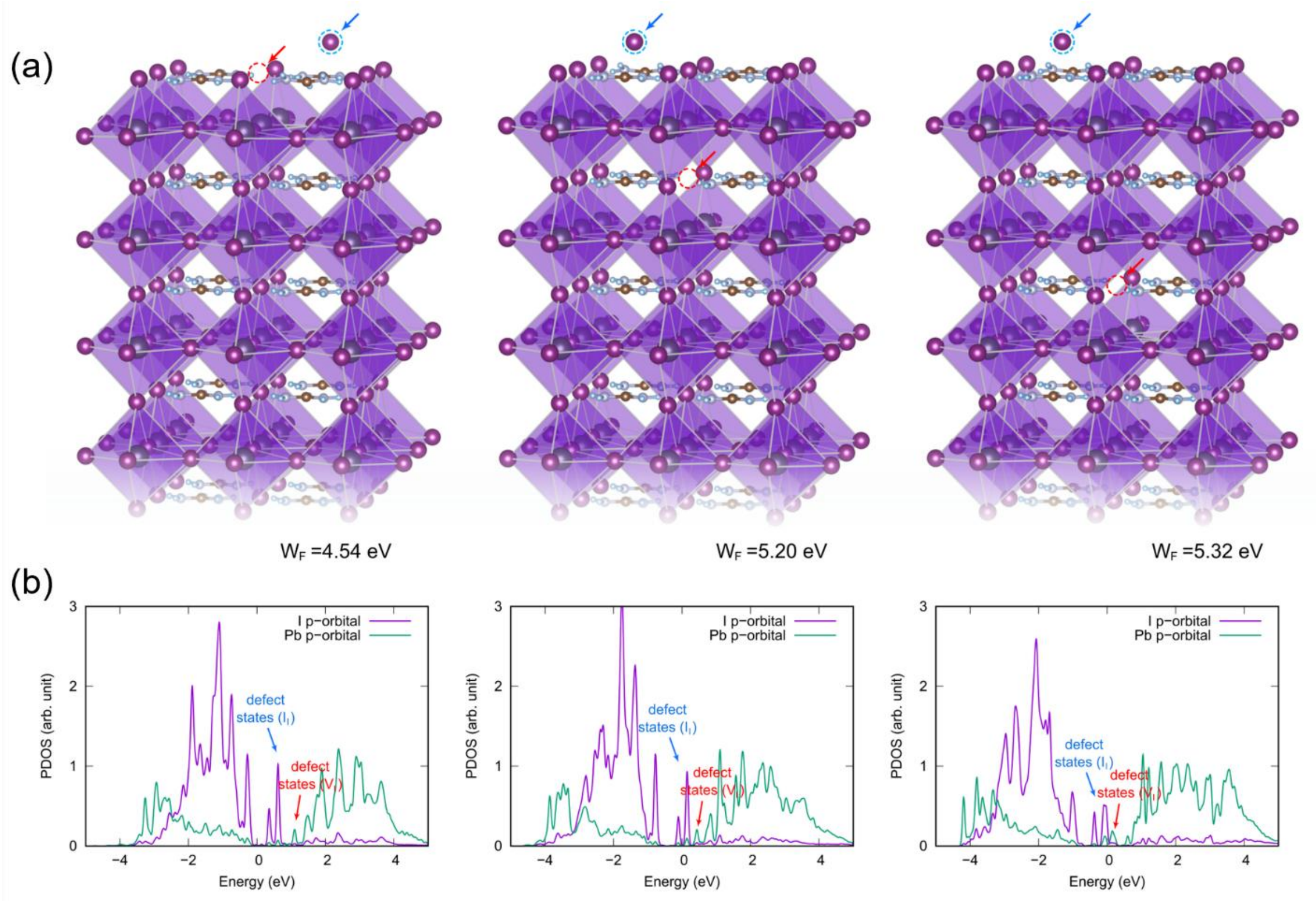

Figure S14. (a) Atomic models of FA-I-terminated $FAPbI_3$ surfaces incorporating an iodine interstitial (I_i) - vacancy (V_I) Frenkel defect pair, where the V_I is located at the first (left), second (middle), and third (right) atomic layers from the surface. The positions of the interstitial iodine atoms (iodine vacancies) are marked with blue (red) arrows. (b) Projected density of states (PDOS) of Pb and I atoms adjacent to the defect sites for each Frenkel pair configuration. The PDOS clearly reveals the simultaneous presence of defect-induced states associated with both V_I and I_i near the Fermi level. As the V_I is positioned deeper below the surface, the vacancy-related donor states are progressively suppressed, while the acceptor-like states originating from the surface-localized I_i become more dominant. This evolution of the defect electronic structure correlates with the systematic increase in the calculated surface work function as the vacancy migrates away from the surface.

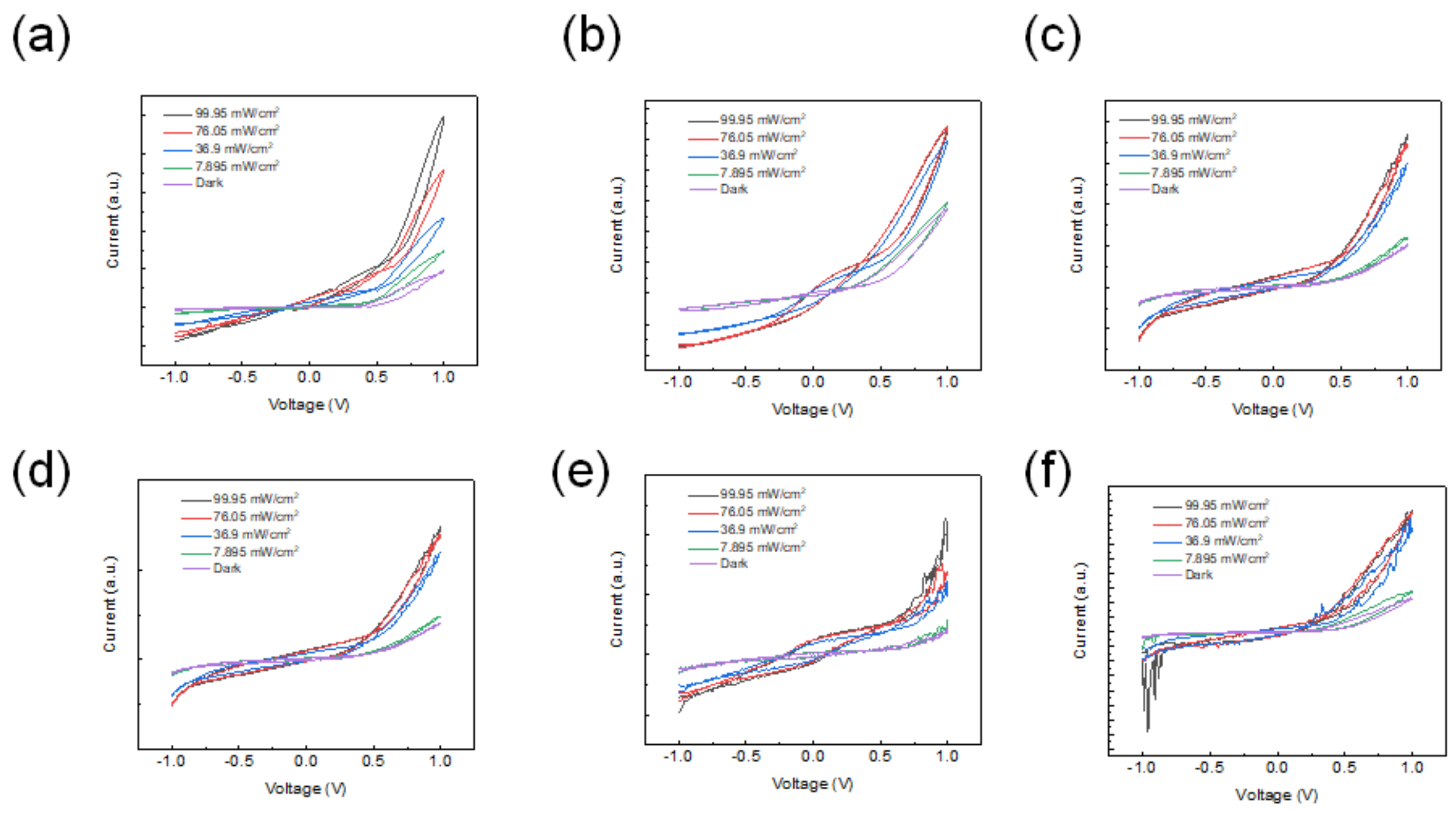

Figure S15. Reproducibility of e-beam-induced lateral homojunction behavior in proof-of-concept perovskite photodetector (PePD) devices with an Au/$FAPbI_3$/Au configuration.

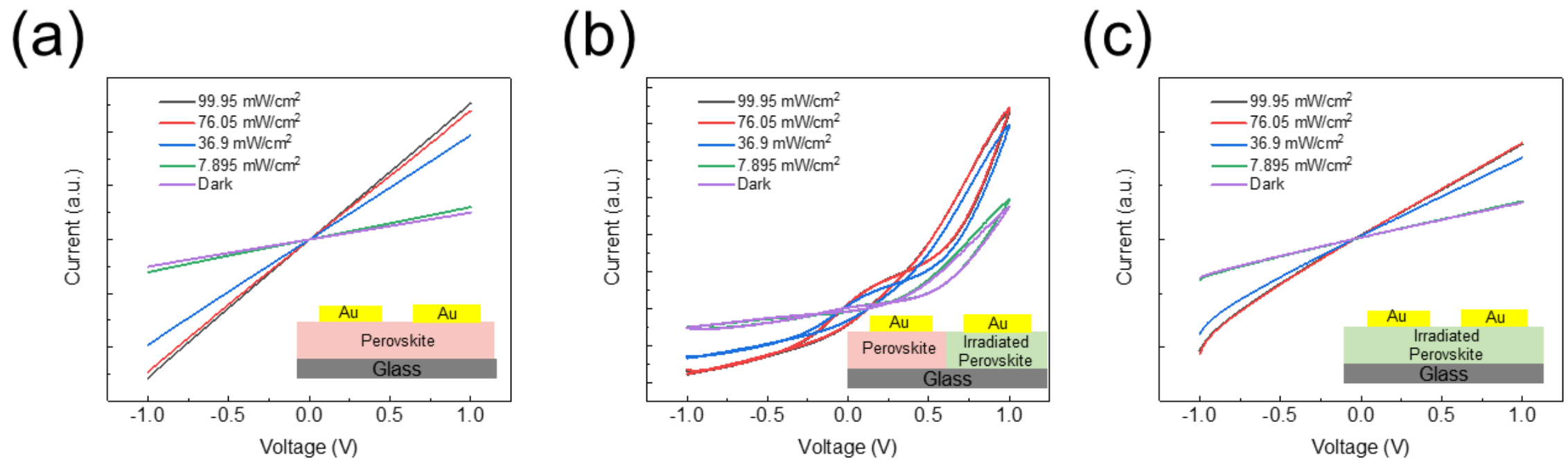

Figure S16. Light-intensity-dependent I-V curves of (a) non-irradiated, (b) half-area-irradiated and full-area-irradiated Au/$FAPbI_3$/Au lateral devices. Each I-V curve was acquired from one representative device for each condition.

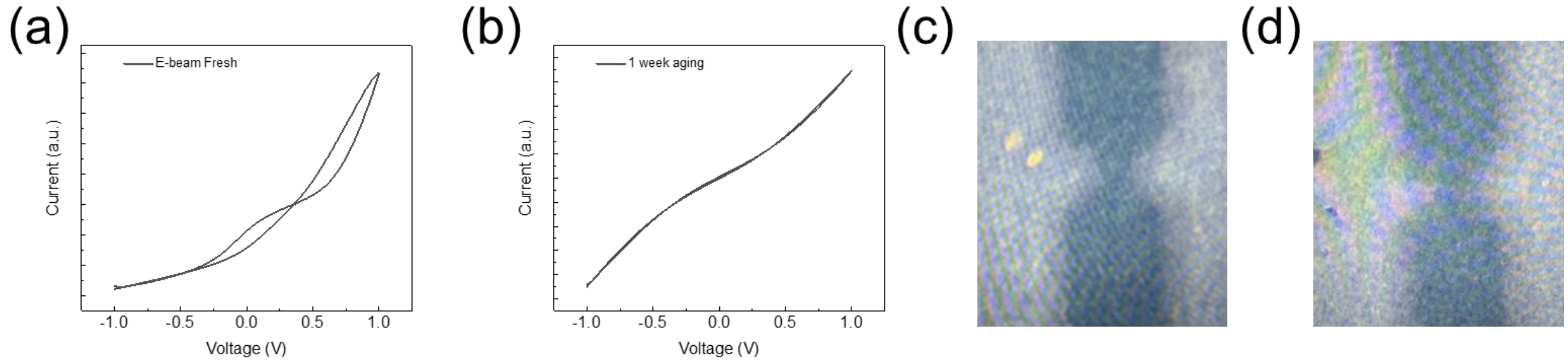

Figure S17. Aging-dependent I-V characteristics of the e-beam-defined Au/$FAPbI_3$/Au planar lateral junction device measured (a) before and (b) after aging under an N_2 atmosphere. Optical microscopy images of the corresponding device (c) before and (d) after aging under an N_2 atmosphere.

Table S1. Experimental reports on electrical doping strategies in perovskite materials

No.	Application		Method	Intermediate	Perovskite	Potential Shift (V)	Ref
1	Solar cell	Intrinsic defects	Composition / Heat treatment	A:B Ratio	$MAPbI_3$	0.80	*(4)* Appl. Phys. Lett. 2014
2	Thin film		Composition	A:B Ratio	$MAPbI_3$ / $FAPbI_3$	0.40	*(5)* J. Phys. Chem. C 2018
3	Thin film		Composition	Excess PbI_2	$MAPbI_3$	0.09	*(6)* Adv. Energy Mater. 2019
4	Solar cell		Composition	A:B Ratio	$MAPbI_3$	0.25	*(7)* J. Phys. Chem. Lett. 2018
5	Solar cell		Heat treatment	interdiffusion	$MAPbI_3$	0.13	*(8)* J. Mater. Chem. A 2014
6	Solar cell		Heat treatment	MA removal	$MAPbI_3$	0.55	*(9)* J. Phys. Chem. C 2015
7	Solar cell		Vapor exposure	I_2	$MAPbI_3$	0.15	*(10)* ACS Energy Lett. 2017
8	Solar cell / Memristor		Biasing	Electric field	$MAPbI_3$	0.22	*(11)* Nat. Mat. 2015
9	Photodetector		Illumination & Biasing	Light & Electric field	$MAPbI_3$	Not specified	*(12)* Chem. Commun. 2015
10	Solar cell		Heat treatment	MA removal	$MAPbI_3$	0.47	*(13)* Sol. RRL 2017
11	Single crystal		Metal-ion interaction	V_I & V_{MA}	$MAPbI_3$	Not specified	*(14)* J. Am. Chem. Soc. 2017
12	Thermodynamic module		Metal-ion interaction	V_I & V_{MA} & V_{Pb}	$MAPbI_3$	Not specified	*(15)* Adv. Electron. Mater. 2021
13	Diode		Oxidation	O_2	$MAPbI_3$	0.50	*(16)* J. Phys. Chem. Lett. 2016
14	Solar cell	Extrinsic defects	Ag / Pb substitution	Ag	$MAPbI_3$	0.92	*(17)* Nano Lett. 2017
15	Field effect transistor		Ag / Pb substitution	Ag	$CsPbBr_3$	0.20	*(18)* ACS Energy Lett. 2019
16	Solar cell		Grain boundary / Interstitial	Li	$MAPbI_3$ / $FACsPbI_3$ / FAMACsPb(IBr)3	0.28 / 0.18	*(19) / (20)* Matter 2020 Eco. Mat. 2023
17	Nano crystal		NCs core injected	Bi	$CsPbBr_3$	0.24	*(21)* J. Am. Chem. Soc. 2017
18	Solar cell		Interstitial	Li	$FACsPb(I_{2.3}Br_{0.7})$	0.15	*(22)* ACS Energy Lett. 2024
19	Solar cell		Interstitial	K	$MAPbI_3$	0.45	*(23)* J. Mater. Chem. A 2017
20	Solar cell		Interstitial	Mg / Sr / Ca	$MAPbI_3$	0.20	*(24)* J. Am. Chem. Soc. 2020

21	Solar cell		Substrate	Cu_2O / NiO/ PEDOT:PSS / FTO / Al_2O_3 / ZnO / TiO_2 / ZrO_2	$MAPbI_3$	1.04	*(25)* Phys. Chem. Chem. Phys., 2014
22	Solar cell		Substrate	NiOx /TiO_2 /C60	$MAPbI_3$	0.60	*(26)* Adv. Mater. Interfaces 2015
23	Thin Film		Substrate	PEIE / ITO / MoO3<br>PEDOT:PSS	$MAPbI_3$	0.50	*(27)* Scientific Reports 2017
24	Photodetector / Thin film transistor		Substrate	MoO_3	$MAPbI_3$	1.10	*(28)* ACS nano 2020
25	Solar cell		Defect reduction	Defect compensation	$MAPbI_3$	0.50	*(29)* Adv. Energy Mater. 2020
26	Thin film		Small molecule	Cobaltocene	$MAPbI_3$	0.62	*(30)* Adv. Electron. Mater. 2018
27	Solar cell	Charge transfer	Small molecule	Mo(tfd-$COCF_3$)$_3$	$FA_{0.85}MA_{0.15}Pb(I_{0.85}Br_{0.15})_3$	0.47	*(31)* Energy Environ. Sci. 2019
28	Single crystal		Small molecule	HZ-TIPS	$CsPbBr_3$	0.40	*(32)* Nano Res, 2019
29	Solar cell		Small molecule	PT-TPA	$FA_xMA_{1-x}PbI_3$	0.29	*(33)* Nat. Commun. 2019
30	Solar cell		Small molecule	F4TCNQ	$MAPbI_3$	0.20	*(34)* Adv. Mater. 2020
31	Field effect transistor / Solar cell		Small molecule	DMBI-2-Th-I	$MAPbI_3$	0.52	*(35)* Adv. Funct. Mater. 2020
32	Photodetector / Field effect transistor		Small molecule	F4TCNQ	$CsPbI_3$	1.29	*(36)* Adv. Mater. 2019
33	Thin film		Defect reduction	Defect compensation	$CsPbBr_3$	0.64	*(37)* ACS Energy Lett. 2022
34	Triboelectric		Biasing	Pb-I Charge density	$FAPbI_3$	0.49	*(38)* ACS Energy Lett. 2021

Table S2. I:Pb ratio of pristine perovskite.

Core level	Peak Area	Relative Sensitivity Factor[39]
I $3d_{3/2}$	1264270	42.42
I $3d_{5/2}$	1842300	
Pb $4f_{5/2}$	446711	33.53
Pb $4f_{7/2}$	470422	
I:Pb ratio	2.68	

Table S3. Photovoltaic parameters of devices measured under forward and reverse scan directions.

n-i-p	Reverse	Forward
Jsc (mA cm^{-2})	24.32 ± 0.14	24.33 ± 0.15
Voc (V)	1.142 ± 0.004	1.142 ± 0.004
Fill Factor	0.783 ± 0.013	0.776 ± 0.010
PCE (%)	21.75 ± 0.37	21.56 ± 0.35
p-i-n	Reverse	Forward
Jsc (mA cm-2)	25.83 ± 0.12	25.84 ± 0.11
Voc (V)	1.151 ± 0.007	1.147 ± 0.006
Fill Factor	0.846 ± 0.004	0.847 ± 0.004
PCE (%)	25.16 ± 0.20	25.12 ± 0.15

Table S4. Average work function of e-beam treated area in perovskite samples under varying acceleration voltages and exposure time.

Ref [4.35 eV]	5 kV	10 kV	15 kV	20 kV	25 kV
3 min	4.45 ± 0.08	4.66 ± 0.09	4.69 ± 0.07	4.72 ± 0.06	4.76 ± 0.07
10 min	4.57 ± 0.07	4.82 ± 0.05	4.84 ± 0.04	4.85 ± 0.04	4.87 ± 0.05
20 min	4.72 ± 0.06	4.83 ± 0.06	4.88 ± 0.04	4.90 ± 0.04	4.93 ± 0.05

Table S5. Average work function of untreated areas in perovskite samples under varying acceleration voltages and exposure time.

Ref [4.35 eV]	5 kV	10 kV	15 kV	20 kV	25 kV
3 min	4.40 ± 0.09	4.41 ± 0.07	4.35 ± 0.07	4.35 ± 0.07	4.37 ± 0.06
10 min	4.41 ± 0.06	4.36 ± 0.06	4.33 ± 0.06	4.33 ± 0.06	4.35 ± 0.07
20 min	4.43 ± 0.06	4.33 ± 0.06	4.35 ± 0.08	4.35 ± 0.07	4.40 ± 0.07

Table S6. Estimated beam current and dose rate for the KPFM irradiation series.

Acceleration voltage (kV)	Emission current (μA)	Estimated beam current (pA)	Dose rate for 40×40 μm^2 ($e^{-}Å^{-2}s^{-1}$)
5	61	55.45–66.55	(2.16–2.60)×10^{-3}
10	58	52.73–63.27	(2.06–2.47)×10^{-3}
15	55	50.00–60.00	(1.95–2.34)×10^{-3}
20	52	47.27–56.73	(1.84–2.21)×10^{-3}
25	50	45.45–54.55	(1.77–2.13)×10^{-3}

Table S7. Total electron dose for the KPFM irradiation series **(e^{-}Å^{-2}).**

Acceleration voltage (kV)	Emission current (μA)	Estimated beam current (pA)	Total dose (e^{-}Å^{-2})		
			Exposure time		
			3 min	10 min	20 min
5	61	55.45–66.55	0.39–0.47	1.30–1.56	2.60–3.12
10	58	52.73–63.27	0.37–0.44	1.23–1.48	2.47–2.96
15	55	50.00–60.00	0.35–0.42	1.17–1.40	2.34–2.81
20	52	47.27–56.73	0.33–0.40	1.11–1.33	2.21–2.66
25	50	45.45–54.55	0.32–0.38	1.06–1.28	2.13–2.55

Table S8. Estimated dose for the E20 device relevant irradiation condition.

Acceleration voltage (kV)	Emission current (μA)	Estimated beam current (pA)	Irradiated area	Exposure time	Dose rate ($e^{-}Å^{-2}s^{-1}$)	Total dose ($e^{-}Å^{-2}$)
25	50	45.45–54.55	~ 40×80 μm2	40 min	(8.87–10.64)×10-4	2.13–2.55

Table S9. Changes in I:Pb ratio with the increasing e-beam exposure time at 5kV.

Core level	Peak Area			Relative Sensitivity Factor[39]
	Pristine	10 min	20 min	
I $3d_{3/2}$	5.87×10^4	3.06×10^4	2.02×10^4	42.42
I $3d_{5/2}$	4.43×10^4	2.07×10^4	1.36×10^4	
Pb $4f_{5/2}$	1.75×10^4	7.91×10^3	4.97×10^3	33.53
Pb $4f_{7/2}$	1.33×10^4	5.95×10^3	3.91×10^3	
I:Pb ratio	2.64	2.93	3.02	

Table S10. Summary of calculated work functions and physical implications for representative defect models in $FAPbI_3$.

Defect model	Calculated work function (eV)	Main electronic/structural feature	Relevance to observed work-function increase
Pristine $FAPbI_3$ surface	4.61	Reference FA-I terminated surface	Baseline
I vacancy, V_I	3.65	Donor-like defect state near CBM; lowers work function	Inconsistent with observed work-function increase
Surface iodine interstitial-like defect, I_i	5.40	Shallow acceptor-like state near VBM; strong work-function increase	Strongly consistent
I_i-V_I Frenkel pair, V_I in 1st layer	4.54	Mixed surface/subsurface defect character	Weak or limited contribution
I_i-V_I Frenkel pair, V_I in 2nd layer	5.20	Enhanced acceptor-like surface character	Consistent
I_i-V_I Frenkel pair, V_I in 3rd layer	5.32	Energetically most favorable among tested Frenkel-pair configurations; surface iodine-rich character retained	Strongly consistent
FA vacancy, V_{FA}	4.26	Does not increase work function; possible organic-cation-loss defect	Unlikely to explain the observed trend
Pb-I vacancy pair, $V_{Pb\text{-}I}$	4.86	Increases work function but causes large structural distortion and has relatively high formation energy	Possible secondary contribution, but unlikely to be dominant

Table S11. Rectification ratios calculated from the devices shown in Figure S16 with different E-beam-irradiation areas at an applied bias of ±1 V.

Conditions	Rectification Ratio (Forward current /reverse current)
No irradiation	1.017
Full-area-irradiation	0.891
Half-area-irradiation	3.201

1. Kresse, G.; Furthmüller, J., *Physical Review B* **1996,** *54* (16), 11169. DOI 10.1103/physrevb.54.11169.
2. Perdew, J.; Burke, K.; Ernzerhof, M., *Physical Review Letters* **1996,** *77, 3865*. DOI 10.1103/PhysRevLett.77.3865.
3. Weller, M.; Weber, O.; Frost, J.; Walsh, A., *The Journal of Physical Chemistry Letters* **2015,** *6* (16), 3209. DOI 10.1021/acs.jpclett.5b01432.
4. Wang, Q.; Shao, Y.; Xie, H.; Lyu, L.; Liu, X.; Gao, Y.; Huang, J., *Applied Physics Letters* **2014,** *105* (16). DOI 10.1063/1.4899051.
5. Paul, G.; Chatterjee, S.; Bhunia, H.; Pal, A. J., *The Journal of Physical Chemistry C* **2018,** *122* (35), 20194-20199. DOI 10.1021/acs.jpcc.8b06968.
6. Euvrard, J.; Gunawan, O.; Mitzi, D. B., *Advanced Energy Materials* **2019,** *9* (47). DOI 10.1002/aenm.201902706.
7. Danekamp, B.; Muller, C.; Sendner, M.; Boix, P. P.; Sessolo, M.; Lovrincic, R.; Bolink, H. J., *J Phys Chem Lett* **2018,** *9* (11), 2770-2775. DOI 10.1021/acs.jpclett.8b00964.
8. Bi, C.; Shao, Y.; Yuan, Y.; Xiao, Z.; Wang, C.; Gao, Y.; Huang, J., *J. Mater. Chem. A* **2014,** *2* (43), 18508-18514. DOI 10.1039/c4ta04007d.
9. Song, D.; Cui, P.; Wang, T.; Wei, D.; Li, M.; Cao, F.; Yue, X.; Fu, P.; Li, Y.; He, Y.; Jiang, B.; Trevor, M., *The Journal of Physical Chemistry C* **2015,** *119* (40), 22812-22819. DOI 10.1021/acs.jpcc.5b06859.
10. Zohar, A.; Levine, I.; Gupta, S.; Davidson, O.; Azulay, D.; Millo, O.; Balberg, I.; Hodes, G.; Cahen, D., *ACS Energy Letters* **2017,** *2* (10), 2408-2414. DOI 10.1021/acsenergylett.7b00698.
11. Xiao, Z.; Yuan, Y.; Shao, Y.; Wang, Q.; Dong, Q.; Bi, C.; Sharma, P.; Gruverman, A.; Huang, J., *Nat Mater* **2015,** *14* (2), 193-8. DOI 10.1038/nmat4150.
12. Frolova, L. A.; Dremova, N. N.; Troshin, P. A., *Chem Commun (Camb)* **2015,** *51* (80), 14917-20. DOI 10.1039/c5cc05205j.
13. Cui, P.; Wei, D.; Ji, J.; Song, D.; Li, Y.; Liu, X.; Huang, J.; Wang, T.; You, J.; Li, M., *Solar RRL* **2017,** *1* (2). DOI 10.1002/solr.201600027.
14. Wu, T.; Mukherjee, R.; Ovchinnikova, O. S.; Collins, L.; Ahmadi, M.; Lu, W.; Kang, N. G.; Mays, J. W.; Jesse, S.; Mandrus, D.; Hu, B., *J Am Chem Soc* **2017,** *139* (48), 17285-17288. DOI 10.1021/jacs.7b10416.
15. Xie, Z.; Feng, K.; Xiong, Y.; Chen, X.; Liang, Y.; Abid, K.; Xu, L., *Advanced Electronic Materials* **2021,** *7* (3). DOI 10.1002/aelm.202001003.
16. Zohar, A.; Kedem, N.; Levine, I.; Zohar, D.; Vilan, A.; Ehre, D.; Hodes, G.; Cahen, D., *J Phys Chem Lett* **2016,** *7* (1), 191-7. DOI 10.1021/acs.jpclett.5b02618.
17. Chen, Q.; Chen, L.; Ye, F.; Zhao, T.; Tang, F.; Rajagopal, A.; Jiang, Z.; Jiang, S.; Jen, A. K.; Xie, Y.; Cai, J.; Chen, L., *Nano Lett* **2017,** *17* (5), 3231-3237. DOI 10.1021/acs.nanolett.7b00847.
18. Zhou, S.; Ma, Y.; Zhou, G.; Xu, X.; Qin, M.; Li, Y.; Hsu, Y.-J.; Hu, H.; Li, G.; Zhao, N.; Xu, J.; Lu, X., *ACS Energy Letters* **2019,** *4* (2), 534-541. DOI 10.1021/acsenergylett.8b02478.
19. Xiao, C.; Zhang, F.; Li, Z.; Harvey, S. P.; Chen, X.; Wang, K.; Jiang, C.-S.; Zhu, K.; Al-Jassim, M., *Matter* **2020,** *2* (1), 261-272. DOI 10.1016/j.matt.2019.10.005.
20. Choi, S. G.; Lee, J. W., *EcoMat* **2023,** *5* (10). DOI 10.1002/eom2.12398.
21. Begum, R.; Parida, M. R.; Abdelhady, A. L.; Murali, B.; Alyami, N. M.; Ahmed, G. H.; Hedhili, M. N.; Bakr, O. M.; Mohammed, O. F., *J Am Chem Soc* **2017,** *139* (2), 731-737. DOI 10.1021/jacs.6b09575.
22. Choi, S.-G.; Jung, S.-K.; Lee, J.-H.; Kim, J.-H.; Zheng, W.; Lee, J.-W., *ACS Energy Letters* **2024,** *9* (11), 5360-5363. DOI 10.1021/acsenergylett.4c02000.
23. Zhao, P.; Yin, W.; Kim, M.; Han, M.; Song, Y. J.; Ahn, T. K.; Jung, H. S., *Journal of Materials Chemistry A* **2017,** *5* (17), 7905-7911. DOI 10.1039/c7ta01203a.
24. Phung, N.; Felix, R.; Meggiolaro, D.; Al-Ashouri, A.; Sousa, E. S. G.; Hartmann, C.; Hidalgo, J.; Kobler, H.; Mosconi, E.; Lai, B.; Gunder, R.; Li, M.; Wang, K. L.; Wang, Z. K.; Nie, K.; Handick, E.; Wilks, R. G.; Marquez, J. A.; Rech, B.; Unold, T.; Correa-Baena, J. P.; Albrecht, S.; De Angelis, F.; Bar, M.; Abate, A., *J Am Chem Soc* **2020,** *142* (5), 2364-2374. DOI 10.1021/jacs.9b11637.
25. Miller, E. M.; Zhao, Y.; Mercado, C. C.; Saha, S. K.; Luther, J. M.; Zhu, K.; Stevanović, V.; Perkins, C. L.; van de Lagemaat, J., *Physical Chemistry Chemical Physics* **2014,** *16* (40), 22122-22130. DOI 10.1039/C4CP03533J.

26. Schulz, P.; Whittaker‐Brooks, L. L.; MacLeod, B. A.; Olson, D. C.; Loo, Y. L.; Kahn, A., *Advanced Materials Interfaces* **2015,** *2* (7). DOI 10.1002/admi.201400532.
27. Olthof, S.; Meerholz, K., *Sci Rep* **2017,** *7*, 40267. DOI 10.1038/srep40267.
28. Meng, Y.; Lai, Z.; Li, F.; Wang, W.; Yip, S.; Quan, Q.; Bu, X.; Wang, F.; Bao, Y.; Hosomi, T.; Takahashi, T.; Nagashima, K.; Yanagida, T.; Lu, J.; Ho, J. C., *ACS Nano* **2020,** *14* (10), 12749-12760. DOI 10.1021/acsnano.0c03101.
29. Noel, N. K.; Habisreutinger, S. N.; Wenger, B.; Lin, Y.-H.; Zhang, F.; Patel, J. B.; Kahn, A.; Johnston, M. B.; Snaith, H. J., *Advanced Energy Materials* **2020,** *10* (4), 1903231. DOI 10.1002/aenm.201903231.
30. Perry, E. E.; Labram, J. G.; Venkatesan, N. R.; Nakayama, H.; Chabinyc, M. L., *Advanced Electronic Materials* **2018,** *4* (7). DOI 10.1002/aelm.201800087.
31. Noel, N. K.; Habisreutinger, S. N.; Pellaroque, A.; Pulvirenti, F.; Wenger, B.; Zhang, F.; Lin, Y.-H.; Reid, O. G.; Leisen, J.; Zhang, Y.; Barlow, S.; Marder, S. R.; Kahn, A.; Snaith, H. J.; Arnold, C. B.; Rand, B. P., *Energy & Environmental Science* **2019,** *12* (10), 3063-3073. DOI 10.1039/c9ee01773a.
32. Arramel; Pan, H.; Xie, A.; Hou, S.; Yin, X.; Tang, C. S.; Hoa, N. T.; Birowosuto, M. D.; Wang, H.; Dang, C.; Rusydi, A.; Wee, A. T. S.; Wu, J., *Nano Research* **2018,** *12* (1), 77-84. DOI 10.1007/s12274-018-2183-9.
33. Wu, W. Q.; Wang, Q.; Fang, Y.; Shao, Y.; Tang, S.; Deng, Y.; Lu, H.; Liu, Y.; Li, T.; Yang, Z.; Gruverman, A.; Huang, J., *Nat Commun* **2018,** *9* (1), 1625. DOI 10.1038/s41467-018-04028-8.
34. Jiang, Q.; Ni, Z.; Xu, G.; Lin, Y.; Rudd, P. N.; Xue, R.; Li, Y.; Li, Y.; Gao, Y.; Huang, J., *Adv Mater* **2020,** *32* (31), e2001581. DOI 10.1002/adma.202001581.
35. Chen, H.; Zhan, Y.; Xu, G.; Chen, W.; Wang, S.; Zhang, M.; Li, Y.; Li, Y., *Advanced Functional Materials* **2020,** *30* (36). DOI 10.1002/adfm.202001788.
36. Gaulding, E. A.; Hao, J.; Kang, H. S.; Miller, E. M.; Habisreutinger, S. N.; Zhao, Q.; Hazarika, A.; Sercel, P. C.; Luther, J. M.; Blackburn, J. L., *Adv Mater* **2019,** *31* (27), e1902250. DOI 10.1002/adma.201902250.
37. Heo, S.; Roh, K.; Zhang, F.; Tignor, S. E.; Bocarsly, A. B.; Kahn, A.; Rand, B. P., *ACS Energy Letters* **2021,** *7* (1), 211-216. DOI 10.1021/acsenergylett.1c02554.
38. Ma, C.; Kim, B.; Kang, D.-H.; Kim, S.-W.; Park, N.-G., *ACS Energy Letters* **2021,** *6* (8), 2817-2824. DOI 10.1021/acsenergylett.1c01233.
39. Ren, X.; Wang, J.; Lin, Y.; Wang, Y.; Xie, H.; Huang, H.; Yang, B.; Yan, Y.; Gao, Y.; He, J.; Huang, J.; Yuan, Y., *Nature Materials* **2024,** *23* (6), 810-817. DOI 10.1038/s41563-024-01876-2.
